# METTL14‐Induced M^6^A Methylation Increases G6pc Biosynthesis, Hepatic Glucose Production and Metabolic Disorders in Obesity

**DOI:** 10.1002/advs.202417355

**Published:** 2025-04-25

**Authors:** Qiantao Zheng, Xiao Zhong, Qianqian Kang, Zhiguo Zhang, Decheng Ren, Yong Liu, Liangyou Rui

**Affiliations:** ^1^ Department of Molecular and Integrative Physiology University of Michigan Medical School Ann Arbor MI 48109 USA; ^2^ Elizabeth Weiser Caswell Diabetes Institute University of Michigan Michigan 48109 USA; ^3^ Department of Infectious Diseases Hunan Key Laboratory of Viral Hepatitis Xiangya Hospital Central South University Changsha 410008 China; ^4^ Department of Medicine University of Chicago Chicago IL 60637 USA; ^5^ College of Life Sciences Wuhan University Wuhan 430072 China; ^6^ Division of Gastroenterology and Hepatology Department of Internal Medicine University of Michigan Medical School Ann Arbor MI 48109 USA

**Keywords:** G6pc, gluconeogenesis, hepatic glucose production, m^6^A, METTL14, obesity, type 2 diabetes, YTHDF1, YTHDF3

## Abstract

METTL14 dimerizes with METTL3 to install N6‐methyladenosine (m^6^A) on mRNA (m^6^A writers). Subsequently, m^6^A readers bind to m^6^A‐marked RNA to influence its metabolism. RNA m^6^A emerges to critically regulate multiple intracellular processes; however, there is a gap in our understanding of m^6^A in liver metabolism. Glucose‐6‐phosphatase catalytic subunit (G6pc) mediates hepatic glucose production (HGP) and serves as the gatekeeper for glycogenolysis and gluconeogenesis; however, G6pc regulation is not fully understood. Here, METTL14 is identified as a posttranscriptional regulator of G6pc. Liver METTL14, METTL3, and m^6^A‐methylated *G6pc* mRNA are upregulated in mice with diet‐induced obesity. Deletion of *Mettl14* decreases, whereas overexpression of METTL14 increases, *G6pc* mRNA m^6^A in hepatocytes in vitro and in vivo. Five m^6^A sites are identified, and disruption of them (*G6pc* ^Δ^ *^5A^*) blocks METTL14‐induced m^6^A methylation of *G6pc* ^Δ^ *^5A^* mRNA. METTL14 increases both stability and translation of *G6pc* but not *G6pc* ^Δ^ *^5A^* mRNA. YTHDF1 and YTHDF3 but not YTHDF2 (m^6^A readers) bind to m^6^A‐marked *G6pc* mRNA to increase its synthesis. Deletion of hepatic *Mettl14* decreases gluconeogenesis in primary hepatocytes, liver slices, and mice. Hepatocyte‐specific restoration of G6pc reverses defective HGP in *Mettl14* knockout mice. These results unveil a METTL14/*G6pc* mRNA m^6^A/G6pc biosynthesis/HGP axis governing glucose metabolism in health and metabolic disease.

## Graphical abstract

It is shown that obesity is associated with increases in hepatic METTL14 and m^6^A methylation of *G6pc* transcript. YTHDF1 and YTHDF3 bind to m^6^A‐marked *G6pc* mRNA to increase its synthesis. Hepatocyte‐specific deletion of *Mettl14* decreases *G6pc* m^6^A methylation, G6pc biosynthesis, and G6pc‐mediated gluconeogenesis, alleviating glucose metabolic defects in obesity. These findings unveil a METTL14/YTHDF1/YTHDF3/m^6^A‐*G6pc*/hepatic glucose production axis safeguarding liver metabolism.

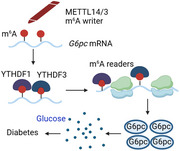

## 1 Introduction

The liver is an essential metabolic organ, and it produces endogenous glucose via glycogenolysis and de novo gluconeogenesis to support metabolic demands and life during fasting, starvation, and exercise. Glycogenolysis rapidly increases hepatic glucose production (HGP) at the early phase of fasting, whereas gluconeogenesis sustains HGP during prolonged starvation. Importantly, excessive HGP causes hyperglycemia and glucose intolerance, hallmarks of type 1 and type 2 diabetes.^[^ ^1^ ^]^ Glucose‐6‐phosphatase converts glucose 6‐phosphate to glucose, a key step of both glycogenolysis and gluconeogenesis.^[^ ^1^ ^]^ Hepatocytes prominently express glucose‐6‐phosphatase catalytic subunit 1 (G6pc) which determines the rate of HGP. Genetic and epigenetic regulation of *G6pc* transcription have been extensively examined; however, mechanisms governing its post‐transcriptional regulation remain elusive. Notably, obesity is a primary risk factor for aberrant HGP and type 2 diabetes, and the obesity epidemic may explain growing prevalence of type 2 diabetes.^[^ ^1^ ^]^ However, it is unclear whether obesogenic factors increase G6pc biosynthesis and HGP by a post‐transcriptional mechanism.

N6‐methyladenosine (m^6^A) is the predominant form of mRNA modifications, and m^6^A methylation profoundly influences pre‐mRNA splicing, RNA decay, mRNA nuclear export, and/or mRNA translation.^[^ ^2^ ^]^ METTL14 (structural subunit) binds to METTL3 (catalytic subunit) and other accessory proteins (WTAP, VIRMA) and forms a methyltransferase complex, called m^6^A writer, to install m^6^A on RNA in a conserved DRACH motif (D = A/G/U, R = A/G, H = A/C/U).^[^ ^3^ ^]^ ALKBH5 and FTO/ALKBH9, referred to as m^6^A erasers, demethylate m^6^A, thereby dynamically influencing m^6^A levels and dynamics.^[^ ^4^ ^]^ A growing number of proteins are recognized to bind to m^6^A‐marked RNA as m^6^A readers, including the YTH family (YTHDF1‐3 and YTHDC1‐2), IGF2BPs, and several members of the HNRNP family.^[^ ^5^ ^]^ The m^6^A readers pivotally regulate the metabolism/fate of client RNAs.^[^ ^2a^ ^]^ Global deletion of *Mettl3* or *Mettl14* results in embryonic death of mice, demonstrating that the METTL14/METTL3/m^6^A pathway is essential for development and survival.^[^ ^6^ ^]^ Adipocyte‐specific deletion of *Mettl14* protects against high fat diet (HFD)‐induced obesity and metabolic disorders by enhancing adipose β adrenergic signaling and lipolysis.^[^ ^7^ ^]^ Likewise, deletion of adipose *Mettl3* also mitigates HFD‐induced obesity.^[^ ^8^ ^]^ These results provide proof of concept evidence that the METTL14/METTL3/m^6^A pathway regulates metabolism and metabolic homeostasis, and raise an intriguing possibility that aberrant activation of the METTL14/METTL3/m^6^A pathway (epitranscriptomic reprogramming) may be a driving force for type 2 diabetes progression. However, there is a knowledge gap in METTL14/METTL3‐elicited epitranscriptomic reprogramming of metabolic pathways in health and disease, including HGP.

In this study, we generated and characterized embryonic or adult‐onset, hepatocyte‐specific *Mettl14* knockout mice. We further characterized mice with liver‐specific overexpression of METTL14. We found that METTL14, in concert with METTL3, installed m^6^A methylation on *G6pc* transcript. In hepatocytes, m^6^A methylation increased G6pc biosynthesis and gluconeogenesis by both suppressing *G6pc* mRNA decay and increasing *G6pc* translation. YTHDF1 and YTHDF3 acted downstream of METTL14 to enhance G6pc biosynthesis. Hepatic METTL14 and METTL3 were upregulated in obese mice and positively correlated with liver levels of *G6pc* mRNA m^6^A methylation and G6pc protein. Deletion of hepatic *Mettl14* decreased HGP and ameliorated HFD‐induced metabolic disorders. These results unravel a previously unrecognized METTL14/*G6pc* mRNA m^6^A/HGP pathway.

## 2 Results

### 2.1 Adult‐Onset and Hepatocyte‐Specific Deletion of *Mettl14* Suppresses HGP, Glucagon Response, and Diet‐Induced Metabolic Disorders

**Figure 1:**
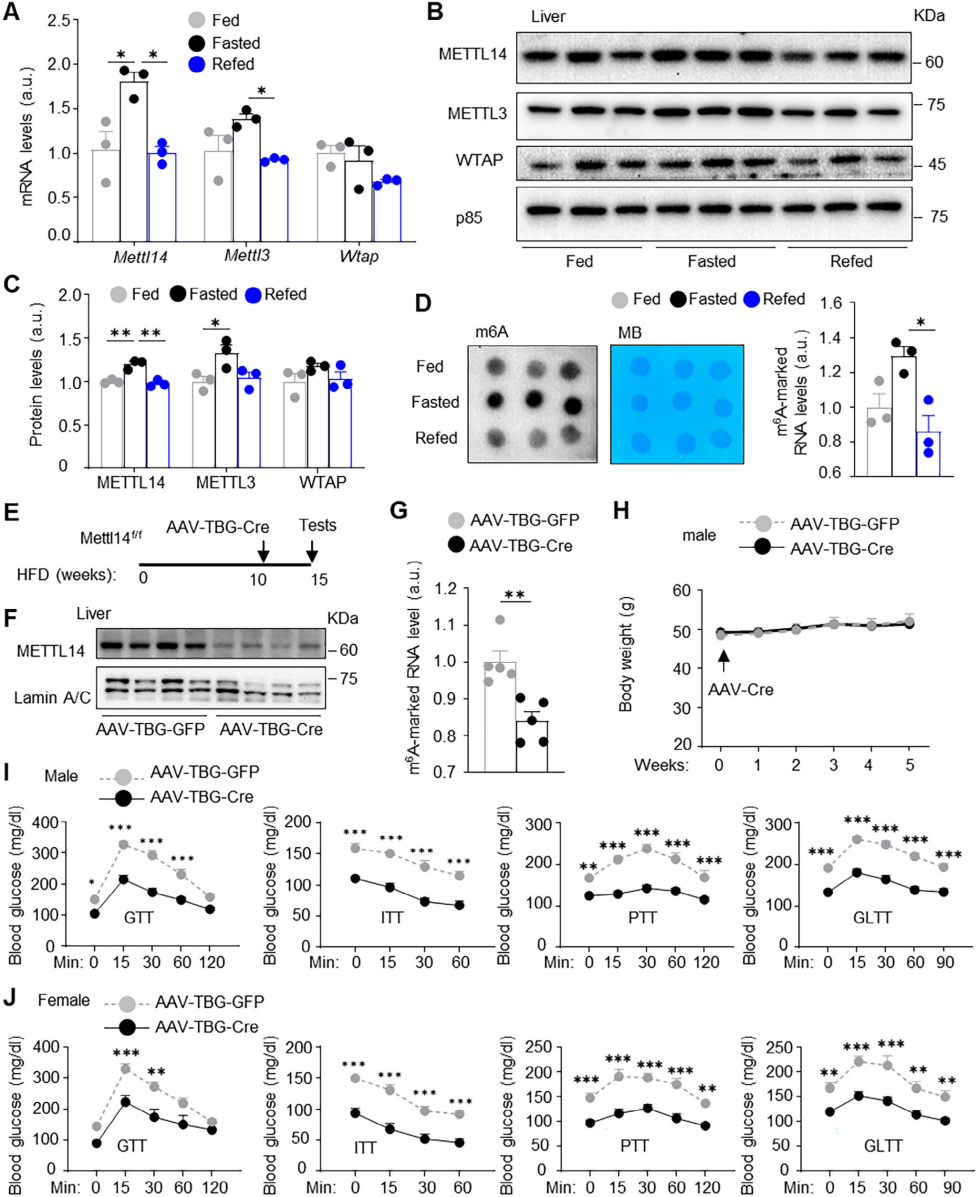
Adult‐onset and hepatocyte‐specific deletion of *Mettl14* improves glucose metabolism. A–D) C57BL/6J males (9 weeks) were fasted overnight and refed for 8 h. A) Liver mRNA levels (normalized to 36B4 levels, *n =* 3 mice per group). a.u.: arbitrary unit. B,C) Liver extracts were immunoblotted with the indicated antibodies. Proteins were normalized to p85 levels (loading control, *n =* 3 mice per group). D) Total liver m^6^A levels were measured using dot blot assays. Total RNA was visualized by methylene blue (MB). m^6^A levels were normalized to MB levels (*n =* 3 per group). E) *Mettl14^f/f^* males or females (8 weeks) were fed a HFD for 10 weeks and transduced with AAV8‐TBG‐GFP or AAV8‐TBG‐Cre vector. F) Male liver nuclear extracts were immunoblotted with the indicated antibodies (6 weeks after AAV transduction). G) Male liver m^6^A levels (dot blot assays in 6 weeks after AAV transduction, *n =* 5 mice per group). H) Male body weight (*n =* 10 per group). I) Male GTT, ITT, PTT, and GLTT in 5–6 weeks post AAV transduction. ITT: *n =* 8 per group; GTT, PTT, and GLTT: *n =* 10 per group. J) Female GTT, ITT, PTT, and GLTT in 5–6 weeks post AAV transduction. GFP: *n =* 7, Cre: *n =* 8. Data are presented as mean ± SEM. *p < 0.05, **p < 0.01, ***p < 0.001, one‐way ANOVA with Tukey's multiple‐comparison test (A–D), two‐sided unpaired *t*‐test (G), and two‐way ANOVA with Šidák's multiple‐comparison test (I,J).

To test if metabolic state influences m^6^A writers, C57BL/6J males were fasted overnight and then refed for 8 h. Liver METTL14 and METTL3 mRNA and protein levels were significantly higher under fasted than in fed conditions; consistently, refeeding substantially decreased hepatic METTL3 and METTL14 levels (**Figure** **1**A–C). In line with these results, total liver RNA m^6^A levels, as measured by m^6^A dot blot assays, were higher in fasted than in fed conditions and significantly decreased by refeeding (Figure 1D). Liver METTL14/METTL3/m^6^A pathways were also activated in obesity (described later). These results support a notion that metabolic signals―nutrients, metabolites, metabolic hormones, neuronal and immune mediators―regulate RNA m^6^A modification pathways in the liver.

To test if RNA m^6^A methylation regulates liver metabolism, we generated and characterized hepatocyte‐specific *Mettl14* knockout mice. *Mettl14^f/f^* mice were fed a HFD for 10 weeks (inducing obesity and metabolic disorders) and transduced with AAV8‐TBG‐Cre vector to specifically delete hepatic *Mettl14* (AAV8‐TBG‐GFP as control) (Figure 1E). *TBG* promoter activation is restricted to hepatocytes.^[^ ^9^ ^]^ We confirmed that METTL14 was substantially decreased in the liver but not skeletal muscle and white adipose tissue (WAT) by AAV8‐TBG‐Cre transduction (Figure 1F and Figure S1A, Supporting Information). Consistently, total liver RNA m^6^A was significantly lower in AAV8‐TBG‐Cre than in AAV8‐TBG‐GFP mice (Figure 1G). Body weight was comparable between AAV8‐TBG‐Cre and AAV8‐TBG‐GFP mice (Figure 1H and Figure S1B, Supporting Information). Five weeks after AAV8 transduction, we assessed glucose clearance and insulin resistance using glucose tolerance tests (GTT) and insulin tolerance tests (ITT), respectively. In GTT, overnight‐fasted blood glucose was significantly lower, and glucose levels were also significantly lower within 60 min post glucose injection in AAV8‐TBG‐Cre than in AAV8‐TBG‐GFP males (Figure 1I). In ITT, blood glucose levels were significantly lower at each point in AAV8‐TBG‐Cre males (Figure 1I). However, percentile of glucose reduction was comparable between AAV8‐TBG‐Cre and AAV8‐TBG‐GFP mice (Figure S1C, Supporting Information). To assess hepatic gluconeogenesis in vivo, gluconeogenic substrate pyruvate was injected into mice in pyruvate tolerance tests (PTT). Blood glucose was substantially lower in AAV8‐TBG‐Cre than in AAV8‐TBG‐GFP males (Figure 1I). We next assessed glucagon sensitivity in glucagon tolerance tests (GLTT), because glucagon is a critical HGP‐stimulating hormone, and its sensitivity is increased in obesity.^[^ ^10^ ^]^ Glucagon increased blood glucose to a significantly less degree in AAV8‐TBG‐Cre than in AAV8‐TBG‐GFP males (Figure 1I,J). Like males, deletion of hepatic *Mettl14* also lowered blood glucose in GTT, ITT, PTT, and GLTT in AAV8‐TBG‐Cre females (Figure 1J). These results suggest that hepatic METTL14‐mediated RNA m^6^A methylation increases HGP and safeguards glucose homeostasis in mice.

### 2.2 Embryonic and Hepatocyte‐Specific Deletion of *Mettl14* Decreases HGP, Glucagon Response, and Blood Glucose

**Figure 2:**
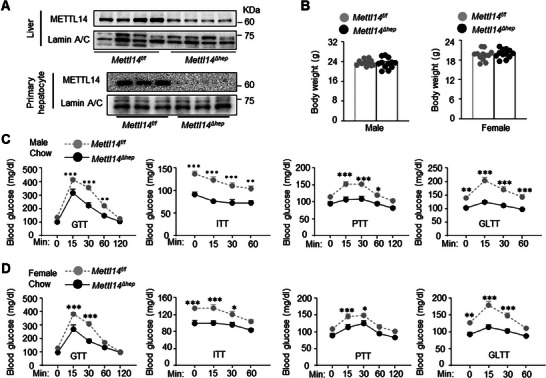
Embryonic and hepatocyte‐specific deletion of *Mettl14* improves glucose metabolism. A) Liver nuclear extracts were immunoblotted with the indicated antibodies (HFD for 10 weeks). Primary hepatocyte nuclear extracts were prepared from chow‐fed mice. B–D) *Mettl14^f/f^* and *Mettl14* ^Δ^ *^hep^* mice were on a chow diet. B) Body weight at 8 weeks of age (*n =* 12 per group). C,D) GTT, ITT, PTT, and GLTT in males (C) and females (D) at 8–10 weeks of age. GTT and ITT: male *Mettl14^f/f^*: *n =* 12, male *Mettl14* ^Δ^ *^hep^*: *n =* 10, female *Mettl14^f/f^*: *n =* 12, female *Mettl14* ^Δ^ *^hep^*: *n =* 12. PTT: male *Mettl14^f/f^*: *n =* 12, male *Mettl14* ^Δ^ *^hep^*: *n =* 8, female *Mettl14^f/f^*: *n =* 8, female *Mettl14* ^Δ^ *^hep^*: *n =* 8. GLTT: *n =* 12 per group. Data are presented as mean ± SEM. *p < 0.05, **p < 0.01, ***p < 0.001, two‐way ANOVA with Šidák's multiple‐comparison test (C,D).

Given that hepatic METTL14 has not been explored in glucose metabolism prior to this study, we further examined its metabolic action by generating and phenotyping embryonic and hepatocyte‐specific *Mettl14* knockout mice (*Mettl14* ^Δ^ *^hep^*). *Mettl14* ^Δ^ *^hep^* mice were generated by crossing *Mettl14^f/f^* mice with *albumin‐Cre* drivers. As expected, METTL14 expression was suppressed specifically in liver but not in skeletal muscle and WAT in *Mettl14* ^Δ^ *^hep^* mice (**Figures** **2**A and S2A, Supporting Information). Consistently, METTL14 was almost undetectable in *Mettl14* ^Δ^ *^hep^* primary hepatocytes (Figure 2A). Body weight was comparable between *Mettl14* ^Δ^ *^hep^* and *Mettl14^f/f^* mice (Figure 2B). We assessed glucose clearance, HGP, insulin resistance, and glucagon sensitivity at 8–10 weeks of age in GTT, ITT, PTT, and GLTT, respectively. Blood glucose was considerably lower in *Mettl14* ^Δ^ *^hep^* males and females than in sex‐matched *Mettl14^f/f^* mice in GTT, ITT, PTT, and GLTT (Figure 2C,D). In ITT, glucose reduction percentile was comparable between *Mettl14* ^Δ^ *^hep^* and *Mettl14^f/f^* mice (Figure S2B, Supporting Information). We next placed *Mettl14* ^Δ^ *^hep^* and *Mettl14^f/f^* mice on HFD for 8 weeks, and body weight was still comparable between *Mettl14* ^Δ^ *^hep^* and *Mettl14^f/f^* mice (Figure S2C, Supporting Information). However, blood glucose remained lower in *Mettl14* ^Δ^ *^hep^* than in *Mettl14^f/f^* mice (both males and females) in GTT, ITT, PTT, and GLTT (Figure S2D,E, Supporting Information).

### 2.3 Hepatocyte‐Specific Deletion of *Mettl14* Mitigates Steatotic Liver Disease

**Figure 3:**
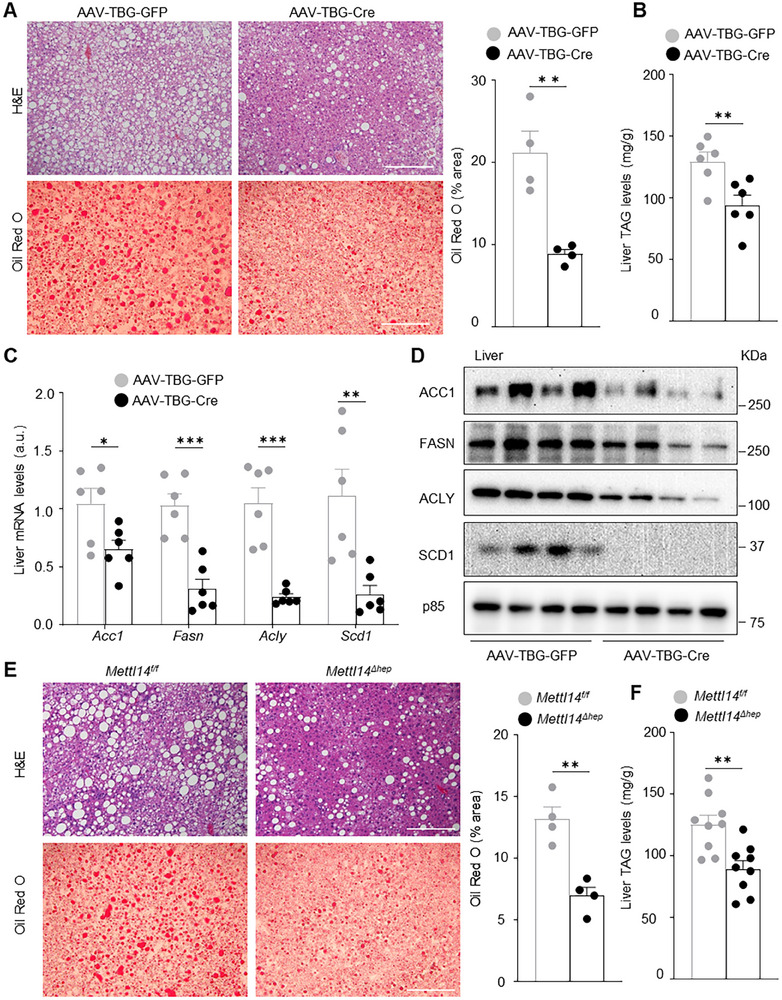
Hepatocyte‐specific deletion of *Mettl14* mitigates HFD‐induced liver steatosis. A–D) *Mettl14^f/f^* males were fed a HFD for 10 weeks and transduced with AAV8‐TBG‐GFP or AAV8‐TBG‐Cre vector. Livers were harvested 6 weeks later. A) H&E and Oil red O staining of liver sections (>3 pairs). Scale bar: 200 µm. B) Liver TAG (normalized to liver weight, *n =* 6 per group). C) Liver mRNA were measured by qPCR and normalized to 36B4 levels (*n =* 6 per group). D) Liver extracts were immunoblotted with the indicated antibodies. E,F) *Mettl14^f/f^* and *Mettl14* ^Δ^ *^hep^* males (10 weeks) were fed a HFD for 9–10 weeks. E) H&E and Oil red O staining of liver sections (>3 pairs). F) Liver TAG (normalized to liver weight, *n =* 9 per group). Data are presented as mean ± SEM. *p < 0.05, **p < 0.01, ***p < 0.001, two‐sided unpaired *t*‐test (A–C, E,F).

Considering a close interplay between glucose and lipid metabolic pathways, we measured liver lipids in *Mettl14* knockout mice. *Mettl14^f/f^* males were fed a HFD for 10 weeks to induce steatotic liver disease (SLD) and then transduced with AAV8‐TBG‐Cre vector to specifically delete hepatic *Mettl14* (AAV8‐TBG‐GFP as control). The liver was harvested 6 weeks after AAV8 transduction. Hepatocyte lipid droplets, as assessed by H&E and Oil Red O staining of liver sections, were smaller and less abundant in AAV8‐TBG‐Cre mice relative to AAV8‐TBG‐GFP mice (**Figure** **3**A). Consistently, liver triacylglycerol (TAG) was significantly lower in AAV8‐TBG‐Cre than in AAV8‐TBG‐GFP mice (Figure 3B). In line with these results, liver mRNA and protein levels of ACC1, FASN, ACLY, and SCD1 (lipogenic enzymes) were lower in AAV8‐TBG‐Cre mice relative to AAV8‐TBG‐GFP mice (Figure 3C,D). Likewise, embryonic deletion of hepatic *Mettl14* also mitigated HFD‐induced SLD in *Mettl14* ^Δ^ *^hep^* mice (on HFD for 10 weeks) (Figure 3E,F). Liver expression of ACC1, FASN, ACLY, and SCD1 was lower in *Mettl14* ^Δ^ *^hep^* than in *Mettl14^f/f^* mice (Figure S3A,B, Supporting Information). Of note, plasma alanine aminotransferase (ALT, a liver injury marker) was slightly higher in *Mettl14* ^Δ^ *^hep^* than in *Mettl14^f/f^* mice on chow diet, but plasma aspartate aminotransferase was normal in *Mettl14* ^Δ^ *^hep^* mice (Figure S3C, Supporting Information). Adult‐onset deletion of hepatic *Mettl14* also slightly increased plasma ALT levels in AAV8‐TBG‐Cre‐transduced *Mettl14^f/f^* mice on HFD (Figure S3D, Supporting Information). In contrast to males, liver lipid levels were comparable both between AAV8‐TBG‐Cre and AAV8‐TBG‐GFP females and between *Mettl14* ^Δ^ *^hep^* and *Mettl14^f/f^* females (Figure S3E–H, Supporting Information). Thus, hepatic METTL14/m^6^A pathways promote SLD in male but not female mice, displaying sexual dimorphism.

### 2.4 Liver‐Specific Overexpression of METTL14 Increases HGP

**Figure 4:**
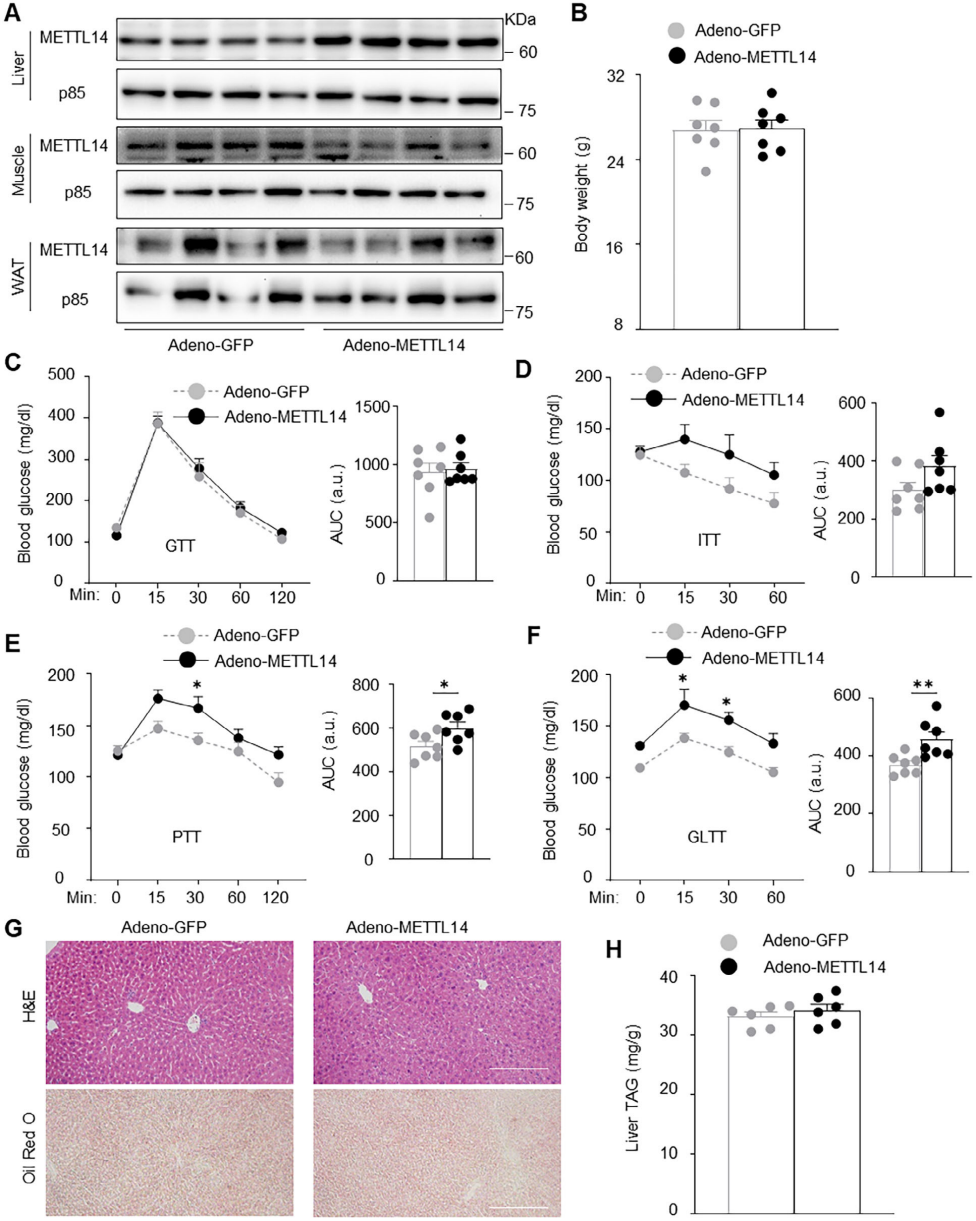
Liver‐specific overexpression of METTL14 increases HGP. C57BL/6J males (10 weeks on chow diet) were transduced with adeno‐METTL14 or adeno‐GFP vector. A) Tissue extracts were immunoblotted with the indicated antibodies (2 weeks post adenoviral transduction). B) Body weight 1 week post adenoviral transduction (*n =* 7 per group). C–F) GTT, ITT, PTT, and GLTT (1 week post adenoviral transduction, *n =* 7 per group). AUC: area under curves. a.u.: arbitrary unit. G,H) Livers were harvested 2 weeks after adenoviral transduction. G) H&E and Oil red O staining of liver sections (>3 pairs). H) Liver TAG (normalized to liver weight, *n =* 6 per group). Data are presented as mean ± SEM. *p < 0.05, **p < 0.01, two‐way ANOVA with Šidák's multiple‐comparison test (the time courses) or two‐sided unpaired *t*‐test (the AUC).

To overexpress METTL14 in the liver, we constructed a METTL14 adenoviral vector (adeno‐METTL14). C57BL/6J male mice were transduced with adeno‐METTL14 or adeno‐GFP vector (control) and metabolically characterized 1 week after adenoviral transduction. METTL14 was modestly overexpressed in the liver but not skeletal muscle and WAT (**Figure** **4**A). Body weight and glucose tolerance were indistinguishable between adeno‐METTL14 or adeno‐GFP group 1 week after adenoviral transduction (Figure 4B,C). In ITT, blood glucose (post insulin injection) and areas under curves (AUC) were higher in adeno‐METTL14 mice but not statistically different from adeno‐GFP mice (Figure 4D). In contrast, in PTT (1 week after adenoviral transduction), blood glucose was significantly higher at 30 min after pyruvate injection in adeno‐METTL14 than in adeno‐GFP mice, and AUC was also significantly higher in adeno‐METTL14 mice (Figure 4E). In GLTT (1 week post adenoviral transduction), blood glucose was significantly higher within 30 min after glucagon injection in adeno‐METTL14 mice relative to adeno‐GFP mice, and AUC was also significantly higher in adeno‐METTL14 mice Figure 4F). Liver lipid levels were similar between adeno‐METTL14 and adeno‐GFP mice (Figure 4G,H). It is likely that METTL14 overexpression alone is insufficient to induce glucose intolerance and liver steatosis.

We observed that METTL14 did not directly influence insulin and glucagon signal transductions. *Mettl14^f/f^* mice (on HFD for 10 weeks) were transduced with AAV8‐TBG‐Cre or AAV8‐TBG‐GFP vector (control). Six weeks later, mice were fasted overnight and stimulated with insulin or glucagon. Insulin stimulated AKT phosphorylation in the liver to a similar degree between AAV8‐TBG‐Cre and AAV8‐TBG‐GFP mice (Figure S4A, Supporting Information). Likewise, glucagon‐stimulated phosphorylation of hepatic CREB was also comparable between AAV8‐TBG‐Cre and AAV8‐TBG‐GFP mice (Figure S4B, Supporting Information). Glucagon‐stimulated phosphorylation of liver CREB was comparable between *Mettl14^f/f^* and *Mettl14* ^Δ^ *^hep^* mice (on HFD for 10 weeks) (Figure S4C, Supporting Information). Liver‐specific overexpression of METTL14 also did not affect glucagon‐stimulated phosphorylation of hepatic CREB (Figure S4D, Supporting Information). These results support a notion that hepatic METTL14 enhances HGP by an insulin/glucagon‐independent mechanism.

### 2.5 METTL14 Directly Increases Gluconeogenesis in Both Liver Slices and Primary Hepatocyte Culture

**Figure 5:**
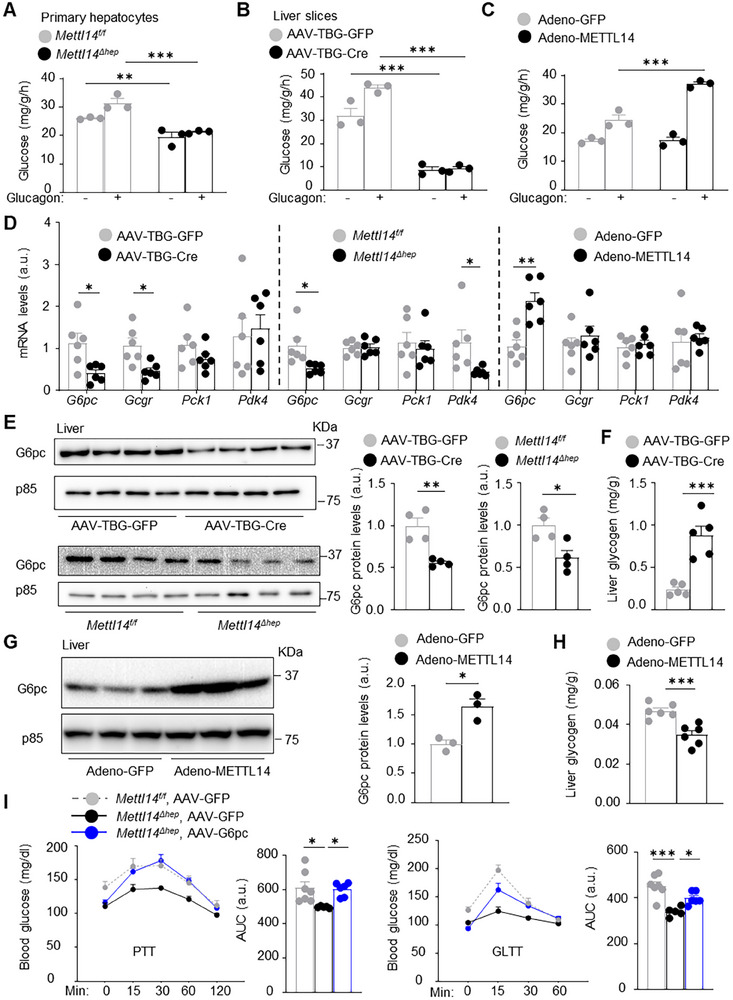
METTL14 cell‐autonomously increases G6pc levels and glucose production in hepatocytes. A) Primary hepatocytes were stimulated with glucagon (10 nm for 5 h) to measure gluconeogenesis using pyruvate and lactate as substrates (normalized to protein levels, *n =* 3 mice per group). B) *Mettl14^f/f^* males (8 weeks) were transduced with AAV8‐TBG‐Cre or AAV8‐TBG‐GFP vector for 6 weeks (on chow diet). Liver slices were treated with glucagon ex vivo (10 nm for 5 h) to measure gluconeogenesis (normalized to protein levels, *n =* 3 mice per group). C) Primary hepatocytes (C57BL/6J mice) were transduced with adeno‐METTL14 or adeno‐GFP vector for 48 h, and then stimulated with glucagon (10 nm) for 5 h. Gluconeogenesis was measured and normalized to protein levels (*n =* 3 per group). D–F) *Mettl14^f/f^* males (on HFD for 10 weeks) were transduced with AAV8‐TBG‐GFP or AAV8‐TBG‐Cre vector. Livers were harvested 6 weeks later. Livers were also harvested from *Mettl14^f/f^* and *Mettl14* ^Δ^ *^hep^* males on HFD for 10 weeks. D) Liver mRNA levels (normalized to 36B4 levels, *n =* 6 mice per group). E) Liver extracts were immunoblotted with anti‐G6pc antibody. G6pc levels were normalized to p85 levels (*n =* 4 mice per group). F) Liver glycogen levels (*n =* 5 mice per group). G,H) C57BL/6J males (10 weeks on chow diet) were transduced with adeno‐METTL14 or adeno‐GFP vector. Liver glycogen was measured 2 weeks later. G) Liver extracts were immunoblotted with anti‐G6pc antibody. G6pc levels were normalized to p85 levels (*n =* 3 mice per group). H) Liver glycogen levels (*n =* 6 mice per group). I) Males (8 weeks on chow diet) were transduced with the indicated AAV8 vectors. PTT and GLTT were performed 3 weeks later. AUC: area under curve. *Mettl14^f/f^*, AAV8‐TBG‐GFP: *n =* 7. *Mettl14* ^Δ^ *^hep^*, AAV8‐TBG‐GFP: *n =* 5. *Mettl14* ^Δ^ *^hep^*, AAV8‐TBG‐G6pc: *n =* 6. Data are presented as mean ± SEM. *p < 0.05, **p < 0.01, ***p < 0.001, two‐way ANOVA with Šidák's multiple‐comparison test (A–C) and two‐sided unpaired *t*‐test (D–H) and one‐way ANOVA with Tukey's multiple‐comparison test (I).

To test if METTL14 cell‐autonomously promotes gluconeogenesis, primary hepatocytes were isolated from *Mettl14^f/f^* and *Mettl14* ^Δ^ *^hep^* mice and stimulated with glucagon. Gluconeogenesis was measured using pyruvate and lactate as substrates. Glucagon stimulated gluconeogenesis in *Mettl14^f/f^* but not *Mettl14* ^Δ^ *^hep^* hepatocytes (**Figure** **5**A). Primary hepatocytes undergo rapid dedifferentiation in cell culture, complicating data interpretation. To circumvent the problem, we assessed gluconeogenesis in precision‐cut liver slices. Liver slices preserve hepatocyte identity and function ex vivo.^[^ ^11^ ^]^ *Mettl14^f/f^* males (8 weeks old) were transduced with AAV8‐TBG‐Cre or AAV8‐TBG‐GFP vector, and liver slices were prepared 6 weeks later. Glucagon stimulated gluconeogenesis in AAV8‐TBG‐GFP but not AAV8‐TBG‐Cre liver slices (Figure 5B). To test if METTL14 overexpression has the opposite effects, primary hepatocytes was transduced with adeno‐METTL14 or adeno‐GFP vector (control) and then stimulated with glucagon. We confirmed METTL14 overexpression in adeno‐METTL14‐transduced hepatocytes (Figure S5A, Supporting Information). METTL14 overexpression significantly increased glucagon‐stimulated gluconeogenesis (Figure 5C). Collectively, these results unveil a previously unrecognized METTL14/RNA m^6^A/gluconeogenesis axis.

### 2.6 METTL14 Cell‐Autonomously Increases G6pc Levels in Hepatocytes

We next set out to identify METTL14 target genes involved in HGP. *Mettl14^f/f^* males were fed a HFD for 10 weeks and then transduced with AAV8‐TBG‐Cre or AAV8‐TBG‐GFP vector (control). Livers were harvested for transcriptomic profiling using RNA‐seq. We identified 1793 upregulated genes and 1208 downregulated genes in METTL14‐deficient livers (1.2 folds, *p* < 0.05). Gene ontology (GO) analysis annotated the differentially expressed genes to multiple intracellular processes, including glycolysis, gluconeogenesis, and non‐alcoholic fatty liver disease (Figure S5B, Supporting Information). Volcano plots and expression heatmaps revealed downregulation of several gluconeogenic genes (e.g., *G6pc*, *Pck1)* and lipogenic genes (e.g., *Fasn*, *Acly*) in *Mettl14*‐null livers (Figure S5C,D, Supporting Information). We confirmed downregulation of liver *G6pc* mRNA in both AAV8‐TBG‐Cre‐transduced *Mettl14^f/f^* mice (relative to AAV8‐TBG‐GFP mice) and *Mettl14* ^Δ^ *^hep^* mice (relative to *Mettl14^f/f^* mice) by qPCR (Figure 5D). Consistently, hepatic G6pc protein was significantly lower in both AAV8‐TBG‐Cre‐transduced *Mettl14^f/f^* mice (relative to AAV8‐TBG‐GFP mice) and *Mettl14* ^Δ^ *^hep^* mice (relative to *Mettl14^f/f^* mice) (Figure 5E). Moreover, G6pc protein was significantly lower in *Mettl14* ^Δ^ *^hep^* than in *Mettl14^f/f^* primary hepatocytes (Figure S5E, Supporting Information). We postulated that G6pc downregulation might enhance glycogen synthesis by increasing glucose 6‐phosphate levels (G6pc substrate and glycogen precursor). In support of the notion, liver glycogen was significantly higher in AAV8‐TBG‐Cre than in AAV8‐TBG‐GFP transduced *Mettl14^f/f^* mice (Figure 5F). To test if METTL14 overexpression has the opposite effect, C57BL/6J males were transduced with adeno‐METTL14 or adeno‐GFP vector, and the liver was harvested 2 weeks later. Liver‐restricted overexpression of METTL14 substantially increased the mRNA and protein levels of hepatic G6pc (Figure 5D–G). Liver glycogen content was significantly lower in adeno‐METTL14 than in adeno‐GFP mice (Figure 5H). We reasoned that restoration of G6pc might rescue HGP in METTL14‐deficient liver. *Mettl14* ^Δ^ *^hep^* mice were transduced with the AAV8‐TBG‐G6pc vector to produce recombinant Gp6c specifically in the liver (AAV8‐TBG‐GFP as control). We confirmed liver expression of recombinant G6pc in AAV8‐TBG‐G6pc‐transduced mice (slightly lower than endogenous G6pc levels) (Figure S5F, Supporting Information). We assessed liver gluconeogenesis (in PTT) and glucagon sensitivity (in GLTT) 3 weeks after AAV8 transduction. In both PTT and GLTT, blood glucose and AUC were lower in *Mettl14* ^Δ^ *^hep^* than *Mettl14^f/f^* mice (Figure 5I). Hepatocyte‐specific restoration of G6pc in *Mettl14* ^Δ^ *^hep^* mice increased blood glucose and AUC to the levels in *Mett14l^f/f^* mice (Figure 5I). In GTT, hepatocyte‐specific restoration of G6pc in *Mettl14* ^Δ^ *^hep^* mice also increased blood glucose and AUC approximately to the level in *Mettl14^f/f^* mice (Figure S5G, Supporting Information). Taken together, these results unveil a previously unrecognized METTL14/G6pc/HGP axis.

### 2.7 METTL14 Increases *G6pc* mRNA Stability and Translation

**Figure 6:**
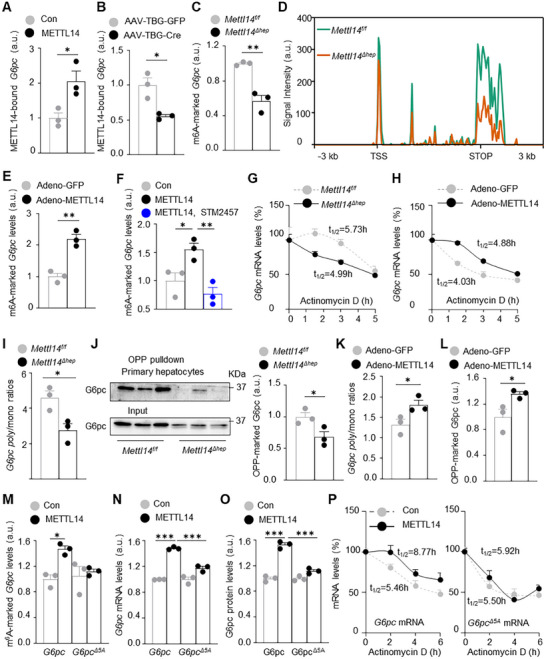
METTL14 m^6^A‐dependently increases *G6pc* mRNA stability and translation in hepatocytes. A) Huh7 cells were cotransfected with *METTL14* and *G6pc* plasmids for 2 days. METTL14‐bound *G6pc* mRNA was measured using METTL14‐linked RIP (*n =* 3 per group). B) *Mettl14^f/f^* males were transduced with AAV8‐TBG‐GFP or AAV8‐TBG‐Cre vector for 6 weeks (on HFD). METTL14‐bound *G6pc* mRNA was measured in the liver using RIP assays (*n =* 3 mice per group). C) Levels of m^6^A‐marked *G6pc* mRNA were measured in primary hepatocytes using MeRIP (normalized to *G6pc* input; *n =* 3 per group). D) Liver m^6^A distribution across the *G6pc* gene between *Mettl14^f/f^* and *Mettl14* ^Δ^ *^hep^* males. E) C57BL/6J mice were transduced with adeno‐METTL14 or adeno‐GFP vector (*n =* 3 per group). 2 weeks later, liver m^6^A‐marked *G6pc* mRNA was measured using MeRIP (normalized to *G6pc* mRNA input). F) Huh7 cells were cotransfected with *METTL14* and *G6pc* plasmids. 12 hours later, cells were treated with STM2457 (5 µg mL^−1^) for 36 h to measure m^6^A‐marked *G6pc* mRNA (normalized to *G6pc* mRNA input, DMSO as control, *n =* 3 per group). G) Primary hepatocytes were treated with actinomycin D to measure *G6pc* mRNA stability (*n =* 3 mice per group). H) Primary hepatocyte culture (C57BL/6J males) was transduced with adeno‐METTL14 or adeno‐GFP vector for 24 h. *G6pc* mRNA decays were assessed using actinomycin D (*n =* 3 mice per group). I) Poly‐bound and mono‐bound *G6pc* mRNA were measured in primary hepatocytes from males (8 weeks). J) OPP assays on primary hepatocytes to assess G6pc translation (normalized to G6pc input; *n =* 3 mice per group). K,L) Primary hepatocyte culture (C57BL/6J males) was transduced with adeno‐METTL14 or adeno‐GFP vector. Poly/mono‐bound *G6pc* mRNA and G6pc translation (OPP assays) were measured 48 and 24 h later, respectively (*n =* 3 mice per group). M–O) Huh7 cells were cotransfected with *METTL14* and *G6pc or G6pc* ^Δ^ *^5A^* plasmids for 2 days. *G6pc* mRNA m^6^A methylations (normalized to *G6pc* input) and *G6pc* mRNA levels (normalized to *GAPDH* levels) were measured. Cell extracts were immunoblotted with anti‐G6pc antibody to measure G6pc levels (normalized to p85 levels, *N* = 3 per group). P) Huh7 cells were cotransfected with *METTL14* and *G6pc or G6pc* ^Δ^ *^5A^* plasmids for 36 h and then treated with actinomycin D. *G6pc* mRNA levels were measured and normalized to the initial values (*n =* 3 per group). Data are presented as mean ± SEM. *p < 0.05, **p < 0.01, ***p < 0.001, two‐sided unpaired *t*‐test (A–C, E, I–L), two‐way ANOVA with Šidák's multiple‐comparison test (G,H,P) and one‐way ANOVA with Tukey's multiple‐comparison test (M–O).

Given that m^6^A methylation influences mRNA stability and/or translation, we postulated that METTL14 might directly promote G6pc biosynthesis. G6pc plasmid was cotransfected with *METTL14* plasmid (empty plasmid as control) into Huh7 cells (human hepatoma line). METTL14 and its bound mRNA were immunopurified with anti‐METTL14 antibody, called RNA immunoprecipitation (RIP), and METTL14‐bound *G6pc* mRNA was measured by qPCR. We readily detected METLL14‐bound *G6pc* mRNA (**Figure** **6**A). To verify METTL14/*G6pc* mRNA interaction in the liver, *Mettl14^f/f^* mice were transduced with AAV8‐TBG‐Cre (deleting hepatic *Mettl14*) or AAV8‐TBG‐GFP vector. Livers were harvested for RIP assays 6 weeks later. METTL14‐bound *G6pc* mRNA was significantly lower in AAV8‐TBG‐Cre than in AAV8‐TBG‐GFP mice (Figure 6B). To test if METTL14 installs m^6^A on *G6pc* transcript, we measured *G6pc* mRNA m^6^A levels in primary hepatocytes using m^6^A‐linked RIP assays (MeRIP). m^6^A‐marked *G6pc* mRNA levels were significantly lower in *Mettl14* ^Δ^ *^hep^* than in *Mettl14^f/f^* hepatocytes (Figure 6C). Liver m^6^A‐marked *G6pc* mRNA levels were also significantly lower in AAV8‐TBG‐Cre than in AAV8‐TBG‐GFP *Mettl14^f/f^* mice (Figure S5H, Supporting Information). To further validate METTL14‐mediated m^6^A in *G6pc* mRNA, we reanalyzed liver MeRIP‐seq datasets generated from *Mettl14^f/f^* and *Mettl14* ^Δ^ *^hep^* mice (GSE185109).^[^ ^12^ ^]^ Deletion of hepatic *Mettl14* markedly reduced m^6^A‐methylated *G6pc* mRNA levels in *Mettl14* ^Δ^ *^hep^* mice (Figure 6D). Notably, m^6^A sites were located around the STOP codon. To verify that METTL14 overexpression has the opposite effect, C57BL/6J males were transduced with adeno‐METTL14 or adeno‐GFP vector (control). Livers were harvested for MeRIP 2 weeks later. Liver‐restricted overexpression of METTL14 dramatically increased m^6^A‐marked *G6pc* mRNA levels (Figure 6E). To test if METTL3 mediates METTL14‐induced m^6^A, Huh7 cells were cotransfected with *G6pc* and *METTL14* plasmids and treated with METTL3 inhibitor STM2457.^[^ ^7^, ^13^ ^]^ METTL14 increased levels of m^6^A‐marked *G6pc* mRNA, and STM2457 completely reversed the METTL14 action (Figure 6F).

To test if METTL14 regulates *G6pc* mRNA decay, *Mettl14* ^Δ^ *^hep^* and *Mettl14^f/f^* primary hepatocytes were treated with transcriptional inhibitor actinomycin D. METTL14 deficiency substantially decreased *G6pc* mRNA stability (Figure 6G). To test if METTL14 overexpression has the opposite effect, primary hepatocytes were transduced with adeno‐METTL14 or adeno‐GFP vector. METTL14 overexpression significantly suppressed *G6pc* mRNA decay (Figure 6H). To test if METTL14 influences *G6pc* mRNA translation in primary hepatocytes, we purified polysomes (poly) and monosomes (mono) with high and low translations, respectively, and measure poly‐ and mono‐associated *G6pc* mRNA by qPCR. The poly/mono ratio was significantly lower in *Mettl14* ^Δ^ *^hep^* than in *Mettl14^f/f^* hepatocytes (Figure 6I). This suggests that METTL14 deficiency decreases G6pc translation. To complement this approach, we directly measured *G6pc* translation using *O*‐propargyl‐puromycin (OPP)‐mediated pulldown assays.^[^ ^7^ ^]^ Newly translated G6pc proteins mark with OPP, and OPP‐tagged G6pc was pulled down with biotin/streptavidin beads and quantified with anti‐G6pc antibody. Nascent G6pc (OPP‐tagged) was significantly lower in *Mettl14* ^Δ^ *^hep^* than in *Mettl14^f/f^* hepatocytes (Figure 6J). In contrast, METTL14 deficiency did not alter p85 translation (Figure S6A, Supporting Information). To verify that METTL14 overexpression has the opposite effect, primary hepatocytes were transduced with adeno‐METTL14 or adeno‐GFP vector. METTL14 overexpression significantly increased both poly/mono *G6pc* mRNA ratio and G6pc translation (OPP assays) (Figure 6K,L and Figure S6B, Supporting Information). As control, METTL14 overexpression did not increase p85 translation (Figure S6C, Supporting Information). Collectively, these results define a previously unrecognized METTL14/*G6pc* mRNA m^6^A/*G6pc* mRNA stability and translation/G6pc biosynthesis/HGP axis.

### 2.8 METTL14 m^6^A‐Dependently Upregulates G6pc Biosynthesis

Given that METTL3 inhibitor STM2457 blocks METTL14‐stimulated m^6^A methylation of *G6pc* mRNA, we postulated that STM2457 might also abrogate the ability of METTL14 to upregulate G6pc synthesis. Huh7 cells were cotransfected with *G6pc* and *METTL14* plasmids and treated with STM2457. Indeed, STM2457 inhibited METTL14 upregulation of G6pc synthesis (Figure S6D,E, Supporting Information). We mapped m^6^A sites by reanalyzing published MeRIP‐seq datasets (Table S1, Supporting Information),^[^ ^14^ ^]^ and identified 28 m^6^A sites across the entire *G6pc* mRNA region (Figure S6F, Supporting Information). To further validate m^6^A sites, we analyzed *G6pc* mRNA sequences using the SRAMP Prediction Server (http://www.cuilab.cn/sramp) and identified A^945^, A^1286^, A^1290^, A^1355^, and A^1362^ as putative m^6^A sites. A^945^, A^1286^, A^1355^, and A^1362^ sites were confirmed in the MeRIP‐seq datasets (Figure S6F, Supporting Information). Next, we replaced A^945^, A^1286^, A^1290^, A^1355^, and A^1362^ with T to generate a *G6pc* ^Δ^ *^5A^* mutant. *G6pc* ^Δ^ *^5A^* or *G6pc* plasmid was cotransfected with *METTL14* plasmid (empty plasmid as control) into Huh7 cells. METTL14 significantly increased m^6^A levels in *G6pc* but not *G6pc* ^Δ^ *^5A^* transcript (Figure 6M). This suggests that A^945^, A^1286^, A^1290^, A^1355^, and A^1362^ are the main m^6^A sites induced by METTL14. Importantly, METTL14 increased the mRNA and protein levels of G6pc but not G6pc^Δ5A^ (Figure 6N,O and Figure S6G, Supporting Information). Consistently, METTL14 increased *G6pc*, but not *G6pc* ^Δ^ *^5A^*, mRNA stability and translation (Figure 6P and Figure S6H, Supporting Information). These results suggest that METTL14 m^6^A‐dependently increases G6pc biosynthesis and HGP in hepatocytes. These results further confirm the METTL14/*G6pc* mRNA m^6^A/G6pc biosynthesis/HGP pathway.

### 2.9 YTHDF1 and YTHDF3 Mediate METTL14‐Induced Upregulation of G6pc Biosynthesis

**Figure 7:**
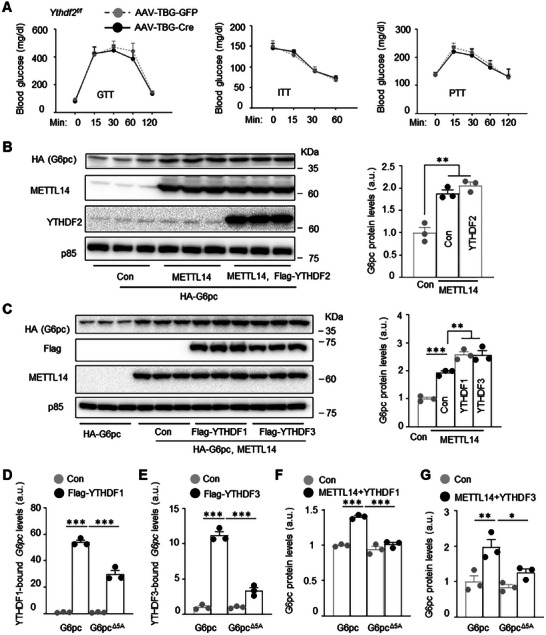
YTHDF1 and YTHDF3 mediate METTL14 upregulation of G6pc biosynthesis. A) *Ythdf2^f/f^* males (HFD for 8 weeks) were transduced with AAV8‐TBG‐Cre or AAV8‐TBG‐GFP vector. PTT, GTT, and ITT were performed 4 weeks later (*n =* 7 mice per group). B) Huh7 cells were cotransfected with *HA‐G6pc*, *YTHDF2*, and *METTL14* plasmids for 2 days. Cell extracts were immunoblotted with the indicated antibodies. HA‐G6pc levels were normalized to p85 levels (*n =* 3 per group). C) Huh7 cells were cotransfected with the indicated plasmids for 2 days. Cell extracts were immunoblotted with the indicated antibodies. HA‐G6pc levels were normalized to p85 levels (*n =* 3 per group). D–G) Huh7 cells were cotransfected with *G6pc*, *G6pc* ^Δ^ *^m6A^*, *Flag‐YTHDF1*, and *Flag‐YTHDF3* plasmids for 2 days (empty plasmid as control). YTHDF1‐bound and YTHDF3‐bound *G6pc* and *G6pc* ^Δ^ *^5A^* mRNAs were measured using RIP assays (normalized to *G6pc* or *G6pc* ^Δ^ *^5A^* mRNA input). Cell extracts were immunoblotted with anti‐G6pc antibody. G6pc levels were normalized to p85 levels (*n =* 3 per group). Data are presented as mean ± SEM. *p < 0.05, **p < 0.01, ***p < 0.001, one‐way ANOVA with Tukey's multiple‐comparison test (A–D) and two‐sided unpaired *t*‐test (E–G).

We next set out to identify m^6^A readers for *G6pc* mRNA. We first tested YTHDF2 because it is a well‐established m^6^A reader to promote mRNA decay.^[^ ^5^, ^15^ ^]^ *Ythdf2^f/f^* mice were fed a HFD for 8 weeks and then transduced with AAV8‐TBG‐Cre (deleting hepatic *Ythdf2*) or AAV8‐TBG‐GFP vector (control). Unexpectedly, PTT, GTT, and ITT were indistinguishable between AAV8‐TBG‐Cre and AAV8‐TBG‐GFP groups (**Figure** **7**A). To test if YTHDF2 influences G6pc synthesis, *HA‐G6pc* plasmid was cotransfected with *METTL14* and *YTHDF2* plasmids into Huh7 cells. Cell extracts were immunoblotted with anti‐HA antibody to measure recombinant G6pc. METTL14 increased G6pc, but YTHDF2 did not further increase G6pc levels (Figure 7B). Thus, it is unlikely that YTHDF2 is a m^6^A reader for *G6pc* mRNA. We then examined YTHDF1 and YTHDF3, which are known to increase mRNA translation.^[^ ^5a,c^ ^]^ *HA‐G6pc* plasmid was cotransfected with *METTL14* and *YTHDF1* (or *YTHDF3*) plasmids into Huh7 cells. Unlike YTHDF2, both YTHDF1 and YTHDF3 significantly increased *HA‐G6pc* expression (Figure 7C). To test if YTHDF1 and YTHDF3 bind to *G6pc* mRNA, *YTHDF1* (or *YTHDF3*) plasmid was cotransfected with *G6pc* or *G6pc* ^Δ^ *^5A^* plasmid into Huh7 cells. YTHDF1‐bound and YTHDF3‐bound *G6pc* mRNA were measured using RIP assays. *G6pc* mRNA abundantly bound to both YTHDF1 and YTHDF3, and abolishing of the five m^6^A sites significantly decreased the binding of *G6pc* ^Δ^ *^5A^* mRNA to YTHDF1 and YTHDF3 (Figure 7D,E). To test if YTHDF1 and YTHDF3 m^6^A‐dependently increase G6pc synthesis, *HA‐G6pc* or *HA‐G6pc* ^Δ^ *^5A^* plasmid was cotransfected with *YTHDF1* or *YTHDF3* plasmid into Huh7 cells. YTHDF1 and YTHDF3 significantly increased G6pc but not G6pc^Δ5A^ levels (Figure 7F,G and Figure S7, Supporting Information). These results reveal a previously unrecognized METTL14/*G6pc* mRNA m^6^A/YTHDF1/YTHDF3/G6pc synthesis/HGP axis.

### 2.10 Obesity Is Associated with Activation of the METTL14/METTL3/*G6pc* mRNA m^6^A/HGP Pathway

**Figure 8:**
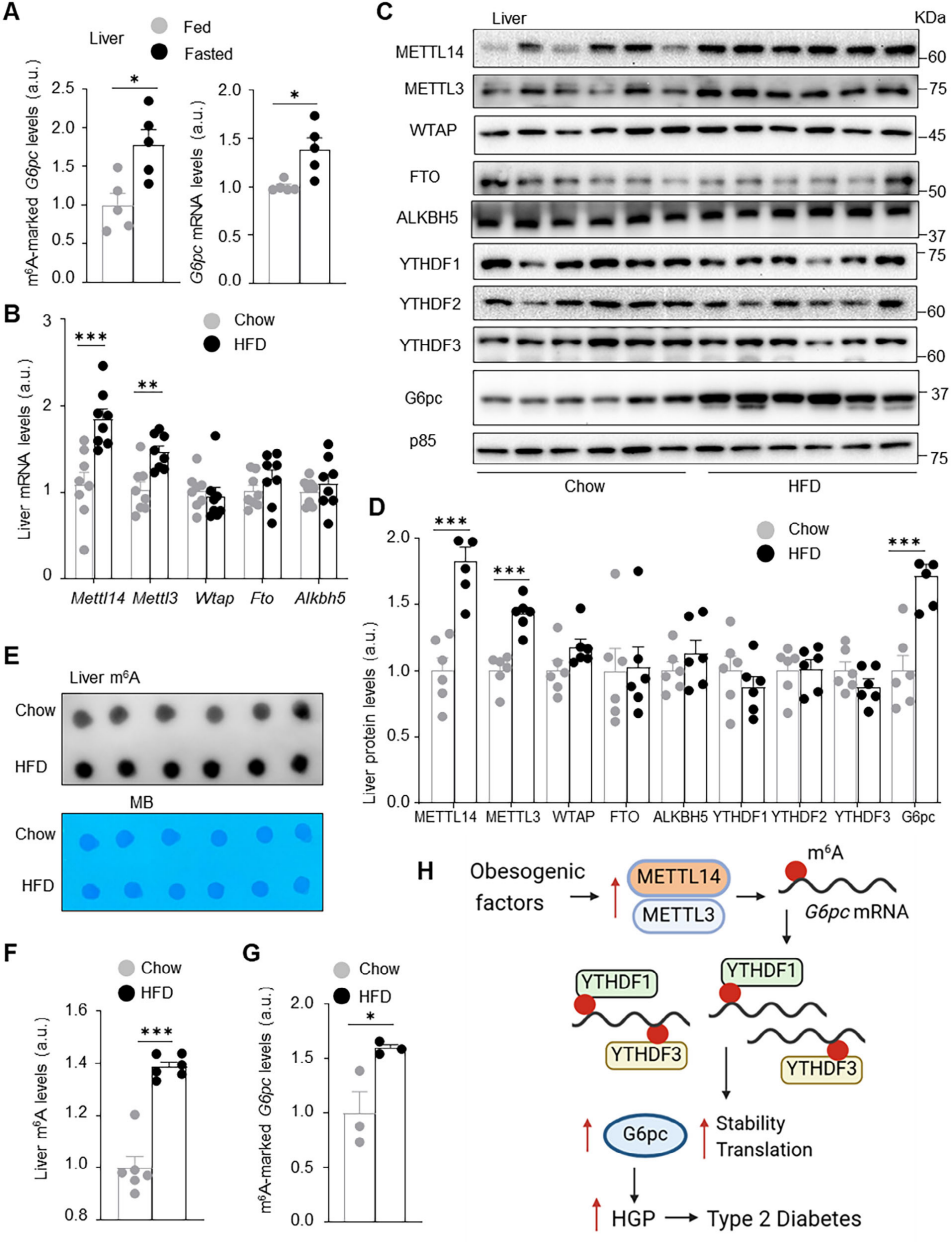
Obesity is associated with activation of the METTL3/METTL14/*G6pc* mRNA m^6^A/G6pc synthesis/HGP axis. A) C57BL/6J males (8 weeks) were fasted for 24 h or randomly fed. Total liver *G6pc* mRNA (normalized to 36B4 levels) and m^6^A‐marked *G6pc* mRNA (normalized to *G6pc* input) were measured (*n =* 5 per group). B–G) C57BL/6J males were fed HFD for 12 weeks (chow diet as control) and fasted overnight to harvest livers. B) Liver RNA abundance (normalized to *36B4* levels, *n =* 8 per group). C,D) Liver extracts were immunoblotted with the indicated antibodies. Protein levels were normalized to p85 levels (*n =* 6 per group). E,F) Total liver m^6^A levels (dot blot assays, *n =* 6 mice per group). G) Liver m^6^A‐marked *G6pc* mRNA was measured by MeRIP (normalized to *G6pc* input, *n =* 6 mice per group). H) Obesogenic factors stimulate upregulation of hepatic METTL14 and METTL3, which in turn install m^6^A on *G6pc* mRNA. YTHDF1 and TRYDF3 bind to m^6^A‐marked *G6pc* mRNA and increase *G6pc* mRNA stability and translation, thereby increasing G6pc synthesis. G6pc increases HGP, thereby promoting hyperglycemia, glucose intolerance, and type 2 diabetes progression. Illustration was obtained from BioRender.com (https://BioRender.com/k70o576). Data are presented as mean ± SEM. *p < 0.05, **p < 0.01, ***p < 0.001, two‐sided unpaired *t*‐test.

Hepatic G6pc and HGP are elevated under fasted conditions. Fasting also increases METTL14 and METTL3 expression in the liver. These prompted us to test if fasting increases m^6^A methylation of *G6pc* mRNA. C57BL/6J males were fasted overnight. Fasting significantly increased m^6^A‐methylated *G6pc* mRNA levels as well as total *G6p c* mRNA levels in the liver (**Figure** **8**A). We postulated that obesogenic factors might upregulate hepatic METTL14 and METTL3 to activate the *G6pc* mRNA m^6^A/G6pc synthesis/HGP pathway, thereby exacerbating type 2 diabetes progression. We placed C57BL/6J males on HFD for 12 weeks and harvested the liver. Both mRNA and protein levels of METTL14 and METTL3 were significantly higher in HFD than in chow diet groups (Figure 8B–D). Consistently, total liver m^6^A levels were significantly higher in HFD than in chow groups (Figure 8E,F). Importantly, liver m^6^A‐marked *G6pc* mRNA levels were significantly higher in HFD‐fed than in chow‐fed mice (Figure 8G). Consequently, hepatic G6pc protein was significantly higher in HFD than in chow groups (Figure 8C,D). These results suggest that obesogenic factors stimulate the METTL14/METTL3/*G6pc* mRNA m^6^A/G6pc synthesis/HGP pathway, thereby augmenting hyperglycemia, glucose intolerance, and type 2 diabetes progression (Figure 8H).

## 3 Discussion

Obesity is associated with aberrantly excessive G6pc‐mediated HGP, promoting type 2 diabetes; however, there is a gap in our understanding of posttranscriptional regulation of G6pc biosynthesis. Here, we unveil a previously unrecognized METTL14/METTL3/*G6pc* mRNA m^6^A/YTHDF1 and YTHDF3/G6pc biosynthesis/HGP pathway governing HGP in health and metabolic disease. METTL14 bound to and installed m^6^A on *G6pc* mRNA. We identified 5 m^6^A sites using the SRAMP prediction server and additionally, mapped 28 m^6^A sites by analyzing publicly available MeRIP‐seq datasets (containing 4 of the 5 sites). We generated *G6pc* ^Δ^ *^5A^* mutant lacking the five m^6^A sites. *G6pc* ^Δ^ *^5A^* mRNA was resistant to METTL14‐induced m^6^A methylation, suggesting that they are the major m^6^A sites. METTL3 inhibitor STM2457 blocked METTL14‐stimulated m^6^A methylation, suggesting that METTL14 partner METTL3 catalyzes m^6^A in *G6pc* mRNA. Functionally, METTL14 increased *G6pc* but not *G6pc* ^Δ^ *^5A^* mRNA stability. Additionally, METTL14 increased G6pc but not G6pc^Δ5A^ translation. Consistently, deletion of hepatic *Mettl14* decreased, whereas hepatocyte‐specific overexpression of METTL14 increased, hepatic G6pc levels in mice. We further showed YTHDF1 and YTHDF3 bound to *G6pc* mRNA to a substantially higher level relative to *G6pc* ^Δ^ *^5A^* mRNA. YTHDF1 increased G6pc but not G6pc^Δ5A^ biosynthesis, YTHDF3 also increased G6pc synthesis to a markedly higher level relative to G6pc^Δ5A^ production. In contrast, hepatic YTHDF2 did not alter G6pc expression and liver metabolism. Based on these findings, we propose that the METTL14/METTL3 complex directly installs m^6^A methylation on *G6pc* mRNA. YTHDF1 and YTHDF3 in turn bind to m^6^A‐marked mRNA to increase G6pc biosynthesis by inhibiting *G6pc* mRNA decay as well as increasing *G6pc* translation (Figure 8H). However, we cannot exclude the possibility that other m6A readers, such as IGF2BP family members, also contribute to upregulation of hepatic G6pc and HGP.

We demonstrated that the METTL14/*G6pc* mRNA m^6^A/G6pc synthesis cascade safeguards HGP. In primary hepatocytes, deletion of *Mettl14* blocked glucagon‐stimulated gluconeogenesis; conversely, overexpression of METTL14 increased gluconeogenesis. In liver slices, METTl14 deficiency markedly decreased gluconeogenesis ex vivo. In animals, hepatocyte‐specific deletion of *Mettl14*, either embryonic or adult‐onset, profoundly impaired gluconeogenesis as assessed by PTT, and hepatocyte‐specific overexpression of METTL14 had the opposite effects. Remarkably, hepatocyte‐specific restoration of G6pc reversed glucagon resistance as well as impaired gluconeogenesis in *Mettl14* ^Δ^ *^hep^* mice. These results uncover, for the first time, a METTL14/*G6pc* mRNA m^6^A/YTHDF1 and YTHDF3/G6pc biosynthesis/HGP pathway safeguarding glucose homeostasis. Hepatic METTL14 and METTL3 were upregulated in obesity. Likewise, liver METTL3 and METTL14 are also upregulated in humans with obesity and diabetes.^[^ ^16^ ^]^ METTL3 and METTL14 in white blood cells are elevated in patients with type 2 diabetes.^[^ ^17^ ^]^ Consistently, total liver RNA m^6^A levels and the level of m^6^A‐marked *G6pc* mRNA were significantly higher in HFD fed mice relative to normal chow‐fed mice. We propose that obesogenic factors upregulate hepatic METTL3 and METTL14, which in turn install m^6^A on *G6pc* mRNA to increase G6pc biosynthesis through increasing both *G6pc* mRNA stability and translation (Figure 8H). Currently, upstream regulators of METTL3 and METTL14 remain elusive. Notably, E3 ligase STUB1 induces ubiquitination and degradation of METTL14.^[^ ^18^ ^]^ RPN11 induces deubiquitylation and upregulation of METTL3.^[^ ^19^ ^]^ It is likely that ubiquitin E3 ligases and deubiquinating enzymes may act upstream to regulate METTL3 and METTL14 activities in obesity. Aberrant elevation of G6pc increases HGP (e.g., glycogenolysis and gluconeogenesis), thereby promoting hyperglycemia, glucose intolerance, and type 2 diabetes progression. Therefore, the METTL14/METTL3/*G6pc* mRNA m^6^A pathway may serve as new therapeutic targets for type 2 diabetes treatment.

We noticed that deletion of hepatic *Mettl14* mitigated HFD‐induced liver steatosis. Likewise, hepatocyte‐specific deletion of *Mettl3* also protects against HFD‐induced liver steatosis.^[^ ^16a^ ^]^ Liver expression of lipogenic enzymes (*Acc1*, *Fasn*, *Acly*, and *Scd1*) was decreased in mice with hepatocyte‐specific deletion of *Mettl14*, which may partially explain protection against liver steatosis. In human hepatocytes, METTL14 increases the m^6^A content of *ACLY* and *SCD1* transcripts and enhances ACLY and SCD1 expressions and lipid accumulation.^[^ ^20^ ^]^ Paradoxically, hepatocyte‐specific deletion of *Mettl3* or *Mettl14* was also reported to augment liver steatosis.^[^ ^21^ ^]^ The reasons for the discrepancy between these studies are unclear. Given that environmental factors profoundly influence metabolic phenotypes, their differences in different animal facility may explain, at least in part, the discrepancy. It is worth mentioning that our findings do not exclude the possibility that the m^6^A writer may act on additional, multiple RNA targets to influence liver metabolism and homeostasis. Moreover, additional m^6^A readers aside from YTHDF1 and YTHDF3 may also mediate METTL14 regulation of G6pc synthesis and HGP. Additional studies are warranted to investigate the hypothesis in the future.

This study has limitations. We detected residual RNA m^6^A methylations in METTL14‐deficient hepatocytes. It is likely that other unknown m^6^A writers may be responsible for the residual m^6^A methylation. We detected a slight increase in plasma ALT in *Mettl14* knockout mice. Other groups have also reported liver injury in mice with hepatocyte‐specific deletion of *Mettl3* or *Mettl14*.^[^ ^12^, ^21^, ^22^ ^]^ Potential contribution of liver injury to HGP remains elusive.

## 4 Experimental Section

### Animals

*Mettl14^f/f^* and *Ythdf2^f/f^* mice (C57BL/6J background) were described previously.^[^ ^23^ ^]^ *Albumin‐Cre* mice (JAX, 0 03574) were also described before.^[^ ^9b^ ^]^ *Mettl14^f/f^* mice were crossed with *albumin‐Cre* drivers to obtain embryonic onset, hepatocyte‐specific *Mettl14* ^Δ^ *^hep^* mice (*Mettl14^f/f^; albumin‐Cre^+/−^*). To generate adult‐onset, hepatocyte‐specific knockout mice, *Mettl14^f/f^* or *Ythdf2^f/f^* mice (8 weeks) were fed HFD for 10 weeks and injected with AAV8‐TBG‐Cre (Addgene #107 787) or AAV8‐TBG‐GFP (control) vector via tail vein (10^11^ viral particles per mouse). AAV8‐transduced mice were on HFD for additional 5–6 weeks and subjected to metabolic tests. Mice were housed on a 12 h light–dark cycle at 25 °C and fed ad libitum a chow diet (9% fat; TestDiet, St. Louis, MO) or HFD (60% fat in calories; Research Diets, New Brunswick, NJ).

### Ethics Statements

Animal research was complied with relevant ethic regulations. Animal experiments were conducted following the protocols approved by the University of Michigan Institutional Animal Care and Use Committee (IACUC).

### Overexpression of METTL14 in Mouse Liver

Human METTL14 cDNA was inserted into a pAdtract‐CMV plasmid at Bgl II and EcoR V sites. pAdtract‐CMV‐METTL14 plasmids were cointroduced with pAdEasy‐1 plasmids into BJ5183 bacteria to generate pAdeay‐1‐METTL14 plasmids. pAdeay‐1‐METTL14 plasmids were transfected into Q293A cells to generate METTL14 adenoviral vector. C57BL/6J males (8 weeks) were fed a chow diet and transduced with METTL14 or GFP adenoviral vectors via tail vein injections (10^11^ viral particles/mouse). It was reported that expression of recombinant proteins is restricted to the liver under these conditions.^[^ ^10^, ^24^ ^]^ Metabolic tests were performed 1 week post adenoviral transduction.

### GTT, ITT, PTT, and GLTT

Mice were fasted 8 h (GTT, PTT) or 6 h (ITT, GLTT), and intraperitoneally injected with glucose (1 or 2 g kg^−1^ body weight, GTT), human insulin (0.4, 0.75, or 1 unit per kg, ITT), pyruvate (1 g kg^−1^, PTT), or glucagon (10 or 15 µg kg^−1^, GLTT). Blood samples were collected from tail veins 0, 15, 30, 60, 90, and 120 min after injection and were used to measure blood glucose levels.

### Liver TAG and Glycogen Levels

Liver samples were homogenized in 1% acetic acid. Lipids were extracted using chloroform/methanol (2:1). Organic fractions were dried in a chemical hood, resuspended in a KOH (3 m)/ethanol solution, incubated at 70 °C for 1 h, and mixed with MgCl_2_ (1 m). Aqueous fractions were used to measure TAG levels using free glycerol reagent (Sigma, Cat#F‐6428).^[^ ^25^ ^]^ Liver tissues were boiled in 30% KOH, and glycogen was precipitated with ethanol and air‐dried. Glycogen pellet was dissolved in water and digested with amyloglucosidase (Sigma, Cat# 10 115) at 42 °C. Glycogen‐derived glucose was measured using glucose liquicolor kit (Stanbio Laboratory, Cat# 1070–125). TAG and glycogen were normalized to liver weight.

### Primary Hepatocyte Cultures and Gluconeogenesis Assays

Primary hepatocytes were isolated from *Mettl14* ^Δ^ *^hep^* and *Mettl14^f/f^* males using type II collagenase (Worthington Biochem, Lakewood, NJ) and grown in William's medium E supplemented with FBS (2%), penicillin (100 units mL^−1^), and streptomycin (100 µg mL^−1^) as described previously.^[^ ^26^ ^]^ To measure gluconeogenesis, hepatocytes were incubated for 5 h in KRB buffer supplemented with lactate (10 mm), pyruvate (5 mm), HEPES (10 mm), BSA (0.6%), and ZnSO_4_ (10 µm) in the presence or absence of glucagon (10 nm). KRB buffer contained NaCl (119 mm), KCI (5 mm), KH_2_PO_4_ (2.6 mm), MgSO_4_ (2.6 mm), CaCl_2_ (2 mm), and NaHCO_3_ (24.6 mm, pH 7.4). Hepatocyte‐produced glucose (released into KRB) was measured using glucose liquicolor kit (ThermoFisher Scientific, Cat# NC9205667) and normalized to hepatocyte protein levels. Primary hepatocytes were isolated from C57BL/6J males and transduced with GFP or METTL14 adenoviral vectors. Gluconeogenesis was measured 48 h after adenoviral transduction.

### Liver Slice Gluconeogenesis

Liver was harvested for 5 h‐fasted mice. A liver block (6 mm diameter) was prepared using Biopsy punch (Henry Schein Incorporated, Cat#9 534 123), mounted on an adaptor using 3% agarose (ThermoFisher Scientific, Cat# BP1356‐500), placed in a cold section buffer constitutively saturated with O_2_: NaCl (137 mm), KCl (5.4 mm), Na_2_HPO_4_ (0.25 mm), glucose (5.5 mm), KH_2_PO_4_ (0.44 mm), CaCl_2_ (1.3 mm), MgSO_4_ (1.0 mm), NaHCO_3_ (4.2 mm), and cut into liver slices (250 µm) using a Leica VT1200 vibratome (Leica Biosystems Nussloch GmbH, Nussloch, Germany). A liver slice was transferred to a porous 70 µm sterile cell strainer (ThermoFisher Scientific, Cat# 22‐363‐548) placed in a 6‐well plate and grown in Williams medium E supplemented with 100 U mL^−1^ penicillin/streptomycin, glutamine (2 mm), insulin–transferrin–selenium X (ITS‐X, at 1X) (ThermoFisher Scientific, Cat# 51 500 056), and FBS (2%), and dexamethasone (100 nm). The plate was placed on a rocker in a CO_2_ incubator (at 37 °C) to generally mix culture medium. Two neighboring wells of the 6‐well plate were connected through a tunnel to facilitate growth medium flow through liver slice surfaces.^[^ ^11^ ^]^ After 18 h incubation, liver slices were washed with PBS and cultured in KRB buffer. After 3 h, liver slices were grown for 5 h in KRB buffer supplemented with lactate (10 mm), pyruvate (5 mm), HEPES (10 mm), BSA (0.6%), and ZnSO_4_ (10 µm) in the presence or absence of glucagon (10 nm). Hepatocyte‐produced glucose was measured and normalized to liver slice protein levels.

### Immunoblotting

Mice were fasted overnight, anesthetized with isoflurane, and injected with insulin (0.75 units per kg body weight) via inferior vena cava. A liver lobe was removed as baseline control before insulin injection. Liver tissues were harvested 5 min later. Mice were fasted overnight and intraperitoneally injected with glucagon and livers were harvested 15 min later. Liver tissue or hepatocyte cultures were homogenized in ice‐cold RIPA buffer: Tris HCl (50 mm, pH 7.5), Nonidet P‐40 (1%), NaCl (150 mm), EGTA (2 mm), Na_3_VO_4_ (1 mm), NaF (100 mm), Na_4_P_2_O_7_ (10 mm), phenylmethylsulfonyl fluoride (1 mm), aprotinin (10 µg mL^−1^), and leupeptin (10 µg mL^−1^). Tissue or cell extracts were resolved by SDS‐PAGE and immunoblotted with the indicated antibodies (Table S2, Supporting Information).

### qPCR

Total RNA was extracted using TRIzol reagents (Invitrogen life technologies, Carlsbad, CA). The first‐strand cDNAs were synthesized using random primers and M‐MLV reverse transcriptase (Promega, Madison, WI). qPCR was performed using Radiant SYBR Green 2X Lo‐ROX qPCR Kits (Alkali Scientific, Pompano Beach, FL), a StepOnePlus RT PCR Systems (Life Technologies Corporation, NY, USA), and respective primers (Table S3, Supporting Information).

### AAV and Expression Plasmids

Human *METTL14* (Addgene, Cat# 53 740) and *YTHDF2* (Addgene, Cat# 52 300) cDNAs were inserted into AAV‐CAG plasmids. Human *YTHDF1* and *YTHDF3* cDNAs were prepared from human Huh7 cells using RT‐PCR and inserted into AAV‐CAG plasmids. Mouse *G6pc1* cDNA was prepared from mouse livers using RT‐PCR and inserted into AAV‐TBG vector. A was replaced with T in the 5 m^6^A sites (5′‐…TGGA^945^CTT…GGGA^1286^CAGA^1290^CAC…TAA…AGGA^1355^CTAGGAA^1362^CAA…‐3′) to generate a *G6pc* ^Δ^ *^5A^* mutant using PCR. DNA insertion was carried out using an In‐Fusion kit (Takar Bio, Cat#638 951). Primers were listed in Table S3 (Supporting Information). AAV8‐TBG‐Cre vector was from Addgene (Cat#107787‐AAV8). The other AAV vectors were generated in the lab.

### RNA‐seq

*Mettl14^f/f^* mice (8 weeks) were fed HFD for 10 weeks and injected with AAV8‐TBG‐Cre (Addgene #107 787) or AAV8‐TBG‐GFP (control) vector via tail vein (10^11^ viral particles per mouse). 8 weeks later, total RNA was extracted by RNeasy Mini Kit (Qiagen Cat#74 104) for RNA‐seq (BGI Genomics, Hong Kong, China). STAR, HTSeq, and DESeq2 software were used to analyze differentially expressed genes between *Mettl14^f/f^* and *Mettl14* ^Δ^ *^hep^* livers. The RNA‐seq data were deposited in the Gene Expression Omnibus (GSE290887).

### m^6^A Dot Blot Assay

Total RNA (1 µg) was spotted on N^+^ membrane, UV‐crosslinked using the auto‐crosslink programs by a XL‐1500 UV crosslinker (Spectrolinker, Westbury, New York), and immunoblotted with anti‐m^6^A antibody. The blots were stained with methylene blue to visualize total RNA. m^6^A levels were quantified and normalized to total RNA.

### m^6^A and METTL14 RIP Assays

Total RNA was extracted from the liver or primary hepatocytes using TRIzol reagents. RNA (10 µg) was incubated at 4 °C with antibodies to m^6^A or METTL14 (1 µL) for 2 h and then with protein‐A agarose beads (10 µL) for an additional 2 h (with rotations). The beads were washed with PBS, and RNA was extracted from the beads using Trizol reagent and used to measure *G6pc* mRNA by qPCR. m^6^A‐marked and METTL14‐bound *G6pc* and G6pc^Δ5A^ RNA were normalized to total *G6pc* and G6pc^Δ5A^ RNA inputs, respectively.

### MeRIP‐seq Dataset Analysis

Published liver MeRIP‐seq datasets generated from *Mettl14^f/f^* and *Mettl14* ^Δ^ *^hep^* mice (GSE185109) were analyzed.^[^ ^12^ ^]^ The reads were aligned to the mm9 reference genome with random chromosomes cleaned by HISAT2 (version 2.2.1) under the parameters ‐p 15‐dta‐no‐mixed‐no‐discordant.^[^ ^27^ ^]^ The uniquely aligned reads were retained, and PCR duplicates were removed using SAMtools (v1.16.1) fixmate and markdup.^[^ ^28^ ^]^ The RefSeq gene annotation files including G6pc were downloaded from the University of California, Santa Cruz (UCSC) browser. Genome coverage bigWig files for the aggregation plots were generated by the deepTools (version 3.5.1) bamCoverage tool with the parameters—normalizeUsing RPKM‐bs 10.^[^ ^29^ ^]^ Total m^6^A sites in *G6pc* mRNA were mapped using online database tool RM2Target and MeRIP‐seq datasets (Table S1, Supporting Information) were published.^[^ ^14^ ^]^

### RNA Stability Assay

Primary hepatocytes were isolated from *Mettl14* ^Δ^ *^hep^* and *Mettl14^f/f^* male littermates and treated with transcription inhibitor actinomycin D (5 µg mL^−1^) for various durations. Total RNA was extracted to measure *G6pc* mRNA levels by qPCR (normalized to *36B4* levels). Results were presented as % of baseline values (absence of actinomycin D treatment) and mRNA half‐life was calculated as previously described.^[^ ^30^ ^]^ Primary hepatocytes were isolated from C57BL/6J males and transduced with METTL14 or GFP adenoviral vectors. 24 h later, hepatocytes were treated with actinomycin D to measure *G6pc* mRNA decay.

### Ribosome Profiling

Primary hepatocytes were isolated from *Mettl14* ^Δ^ *^hep^* and *Mettl14^f/f^* male littermates, washed with ice‐cold PBS supplemented with cycloheximide (100 µg mL^−1^), and collected by centrifugation at 1000 × *g* for 5 min at 4 °C, and lysed in a lysis buffer: KCl (50 mm), Tris HCl (20 mm, pH 7.4), MgCl_2_ (10 mm), Triton X‐100 (1%), 1,4‐dithiothreitol (1 mm), sodium deoxycholate (0.5% w/v), cycloheximide (100 µg mL^−1^), Na_4_P_2_O_7_ (10 mm), benzamidine (1 mm), aprotinin (10 µg mL^−1^), leupeptin (10 µg mL^−1^), phenylmethylsulfonyl fluoride (1 mm), and RNasin. Hepatocyte extracts were centrifuged twice at 2000 × *g* for 5 min and at 13000 × *g* for 5 min. A 7–47% w/v sucrose gradient solution, containing KCl (50 mm), MgCl_2_ (10 mm), Tris HCl (20 mm, pH7.4) was prepared in an ultracentrifugation tube (Backman, 342 413) using a Gradient Maker (C.B.S. Scientific, GM‐20). Cell extracts (6 mL) were loaded on the top of sucrose gradient solution (6 mL) and ultracentrifuged at 260808 × *g* (39000 rpm, Beckman SW 41 rotor) for 90 min in the slow acceleration mode. Consecutive fractions (200 µL per fraction) were collected (total 60 fractions), and their optical absorbance (260 nm) was measured by Synergy 2 microplate reader (BioTek Instruments) to verify monosome and polysome separation. Fractions 1–12 were combined and defined as the monosome pool while fractions 13–60 were combined as the polysome pool. Total RNA was extracted from the two pools to measure individual transcript abundance by qPCR, and polysome‐bound/monosome‐bound mRNA ratios were calculated. Primary hepatocytes were isolated from C57BL/6J males and transduced with GFP or METTL14 adenoviral vectors, and ribosome profiling was conducted 48 h later.

### OPP‐Mediated Pulldown Assay

A previously‐described method was followed.^[^ ^7^ ^]^ Briefly, primary hepatocytes were isolated from *Mettl14* ^Δ^ *^hep^* and *Mettl14^f/f^* male littermates and incubated for 3 h at 37 °C with OPP (30 µm, Click Chemistry Tools, 1407‐5) in growth medium, washed with cold PBS, and lysed in a RIPA buffer. OPP‐tagged nascent proteins in hepatocyte extracts were conjugated with biotin using biotin picolyl azide (Click Chemistry Tools, 1167‐5) using a Click‐and‐Go protein reaction buffer Kit (Click Chemistry Tools, 1262) according to the manufacturer's instructions. The extracts were precipitated with five volumes of cold acetone overnight at −20 °C and centrifuged at 4000 *× g* at 4 °C for 10 min. The pellets were washed with cold methanol and resuspended in PBS containing SDS (1%). The suspension (500 µg proteins) was incubated with streptavidin magnetic beads (Click Chemistry Tools, 1497‐1) at 4 °C overnight with gentle rotations. The beads were washed with cold PBS containing NP40 (1%) and SDS (0.1%) and boiled in 2× Laemmli sample buffer for 10 min to elute OPP‐tagged nascent proteins. OPP‐tagged proteins were quantified by immunoblotting using the indicated antibodies. Primary hepatocytes were isolated from C57BL/6J males and transduced with METTL14 or GFP vectors. OPP assays were performed 24 h later.

### Statistical Analysis

Data were presented as means ± SEM. Differences between two groups were analyzed by 2‐tailed Student's *t*‐test. Comparisons between more than two groups were analyzed by one‐way ANOVA/Tukey's post hoc test using GraphPad Prism 9. Comparisons between two or more groups with more than one variable were analyzed by two‐way ANOVA/Šidák's post hoc test using GraphPad Prism 9. A *P* value less than 0.05 was considered statistically significant.

## Conflict of Interest

The authors declare no conflict of interest.

## Author Contributions

Q.Z. and X.Z. conducted the experiments; Q.Z., X.Z., Q.K., Z.Z., and L.R. analyzed the data; D.R. provided *Mettl14^f/f^* and *Ythdf2^f/f^* mice; Q.Z. and L.R. designed the experiments and wrote the paper; and Q.Z., X.Z., Q.K., D.R., Z.Z., Y.L., and L.R. edited the paper.

## Supporting information



Supporting Information

## Data Availability

The data that support the findings of this study are available in the supplementary material of this article.
