# Supplementary material for: METTL14‐Induced M6A Methylation Increases G6pc Biosynthesis, Hepatic Glucose Production and Metabolic Disorders in Obesity
Source: Adv Sci (Weinh). 2025 Apr 25;12(22):2417355. doi: 10.1002/advs.202417355 (PMC12165098; doi:10.1002/advs.202417355)
Supplement: Supplementary file 1 — Supporting Information [file ADVS-12-2417355-s001.pdf]

ADVANCED SCIENCE
Open Access

Supporting Information

for *Adv. Sci.*, DOI 10.1002/advs.202417355

METTL14-Induced M^6A Methylation Increases G6pc Biosynthesis, Hepatic Glucose Production and Metabolic Disorders in Obesity

*Qiantao Zheng, Xiao Zhong, Qianqian Kang, Zhiguo Zhang, Decheng Ren, Yong Liu and Liangyou Rui**

Supplementary Information

METTL14-induced m^{6}A methylation increases G6pc biosynthesis, liver glucose production and metabolic disorders in obesity

Qiantao Zheng[1,2], Xiao Zhong[1,3], Qianqian Kang[1,2], Zhiguo Zhang[1,2], Decheng Ren[4], Yong Liu[5], Liangyou Rui[1,2,6*]

[1]Department of Molecular & Integrative Physiology, University of Michigan Medical School, Ann Arbor, Michigan 48109, USA

[2]Elizabeth Weiser Caswell Diabetes Institute, University of Michigan, Michigan 48109, USA

[3]Department of Infectious Diseases, Hunan Key Laboratory of Viral Hepatitis, Xiangya Hospital, Central South University, Changsha 410008, China

[4]Department of Medicine, University of Chicago, Chicago, Illinois 60637, USA

[5]College of Life Sciences, Wuhan University, Wuhan 430072, China

[6]Division of Gastroenterology and Hepatology, Department of Internal Medicine, University of Michigan Medical School, Ann Arbor, Michigan 48109, USA

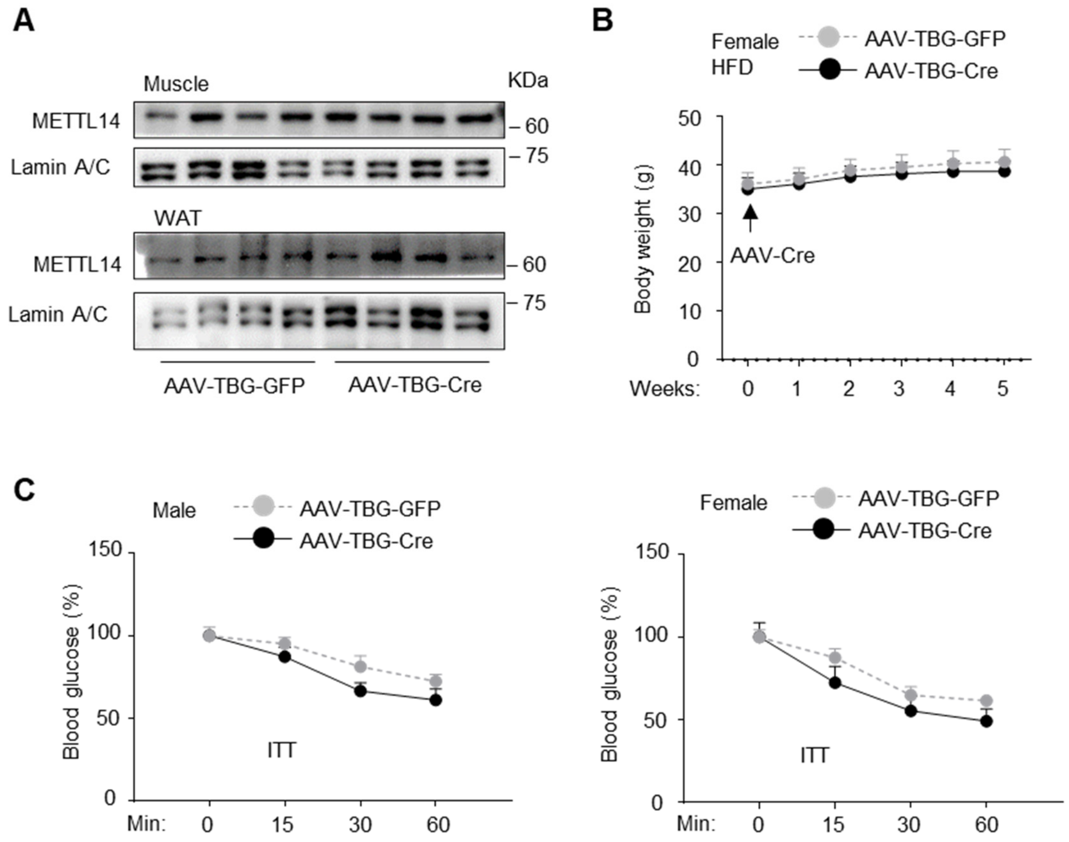

Supplemental Figure 1. Adult-onset and hepatocyte-specific deletion of *Mettl14* does not alter body weight. *Mettl14*$^{f/f}$ male and female males (8 weeks) were fed a HFD for 10 weeks and then transduced with AAV8-TBG-GFP or AAV8-TBG-Cre vector via tail vein injections. **(A)** Nuclear extracts from male skeletal muscle and WAT were immunoblotted with the indicated antibodies (6 weeks after AAV transduction). **(B)** Female body weight. **(C)** ITT (normalized to initial values) in 5 weeks after AAV transduction. Data are presented as mean ± SEM.

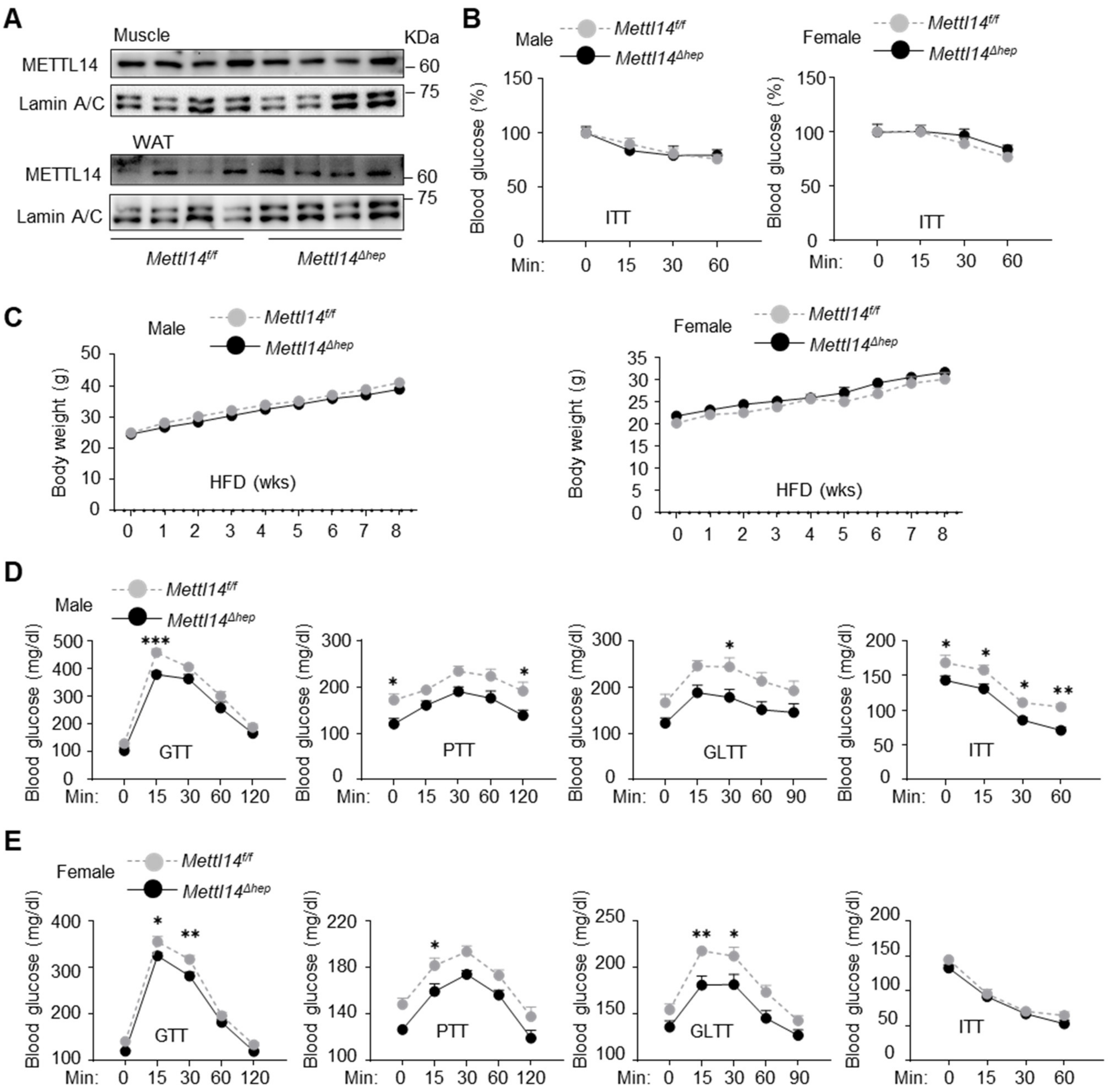

Supplemental Figure 2. Embryonic and hepatocyte-specific deletion of *Mettl14* mitigates HFD-induced metabolic disorders. (A) Nuclear extracts of skeletal muscle and WAT were immunoblotted with the indicated antibodies. **(B)** ITT at 9 weeks of age (on chow diet and normalized to initial values). Male: *Mettl14*$^{f/f}$: n=12, *Mettl14*$^{\Delta hep}$: n=10; female: n=12 per group. **(C-E)** *Mettl14*$^{f/f}$ and *Mettl14*$^{\Delta hep}$ male and female mice were fed a HFD at 10 weeks of age. **(C)** Body weight (male: n=10 per group; female: n=9 for *Mettl14*$^{f/f}$ and n=8 for *Mettl14*$^{\Delta hep}$). **(D-E)** GTT, PTT, GLTT, and ITT were performed in male (**D**, n=9 for *Mettl14*$^{f/f}$ and n=8 for *Mettl14*$^{\Delta hep}$) and female (**E**, for GTT and GLTT, n=10 for *Mettl14*$^{f/f}$ and n=9 for *Mettl14*$^{\Delta hep}$, for PTT and ITT, n=12 for *Mettl14*$^{f/f}$ and n=10 for *Mettl14*$^{\Delta hep}$) from 9 to 10 weeks post HFD. Data are presented as mean ± SEM. *p<0.05, **p<0.01, ***p<0.001, two-way ANOVA with Šidák's multiple-comparison test (**D-E**).

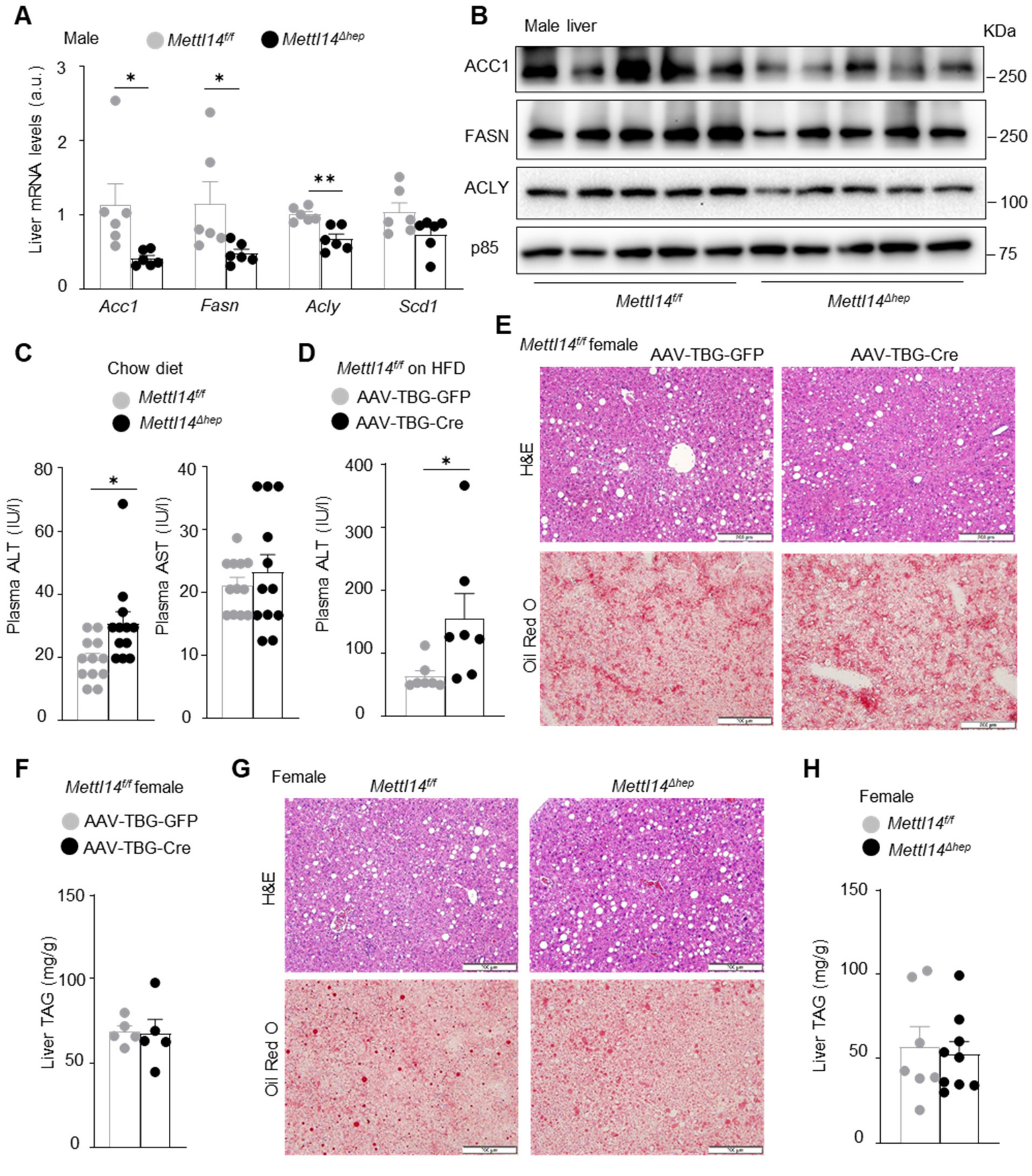

Supplemental Figure 3. Hepatocyte-specific deletion of *Mettl14* ameliorates HFD-induced liver steatosis in males. (A-B) *Mettl14*$^{f/f}$ and *Mettl14*$^{\Delta hep}$ males (10 weeks) were fed a HFD for 10 weeks. **(A)** Liver mRNA levels were measured by qPCR and normalized to 36B4 levels (n=6 per group). **(B)** Liver extracts were immunoblotted with the indicated antibodies (n=5 per group). **(C)** Plasma ALT levels were measured between *Mettl14*$^{f/f}$ and *Mettl14*$^{\Delta hep}$ males on chow diet (9 weeks) (n=12 per group). **(D)** *Mettl14*$^{f/f}$ males (8 weeks) were fed a HFD for 10 weeks and then transduced with AAV8-TBG-GFP or AAV8-TBG-Cre vectors. Plasma ALT levels were measured 6 weeks later (n=7 per group). **(E-F)** *Mettl14*$^{f/f}$ females (8 weeks) were fed a HFD for 10 weeks

and then transduced with AAV8-TBG-GFP or AAV8-TBG-Cre vectors. **(E)** Representative H&E and Oil red O staining of liver sections (>3 pairs). Scale bar: 200 µm. **(F)** Liver TAG levels (normalized to liver weight, n=6 per group). **(G-H)** *Mettl14$^{f/f}$* and *Mettl14$^{\Delta hep}$* females (10 weeks) were fed a HFD for 10 weeks. **(G)** Representative H&E and Oil red O staining of liver sections (>3 pairs). **(H)** Liver TAG levels (normalized to liver weight, n=6 per group). *p<0.05, two-sided unpaired *t*-test.

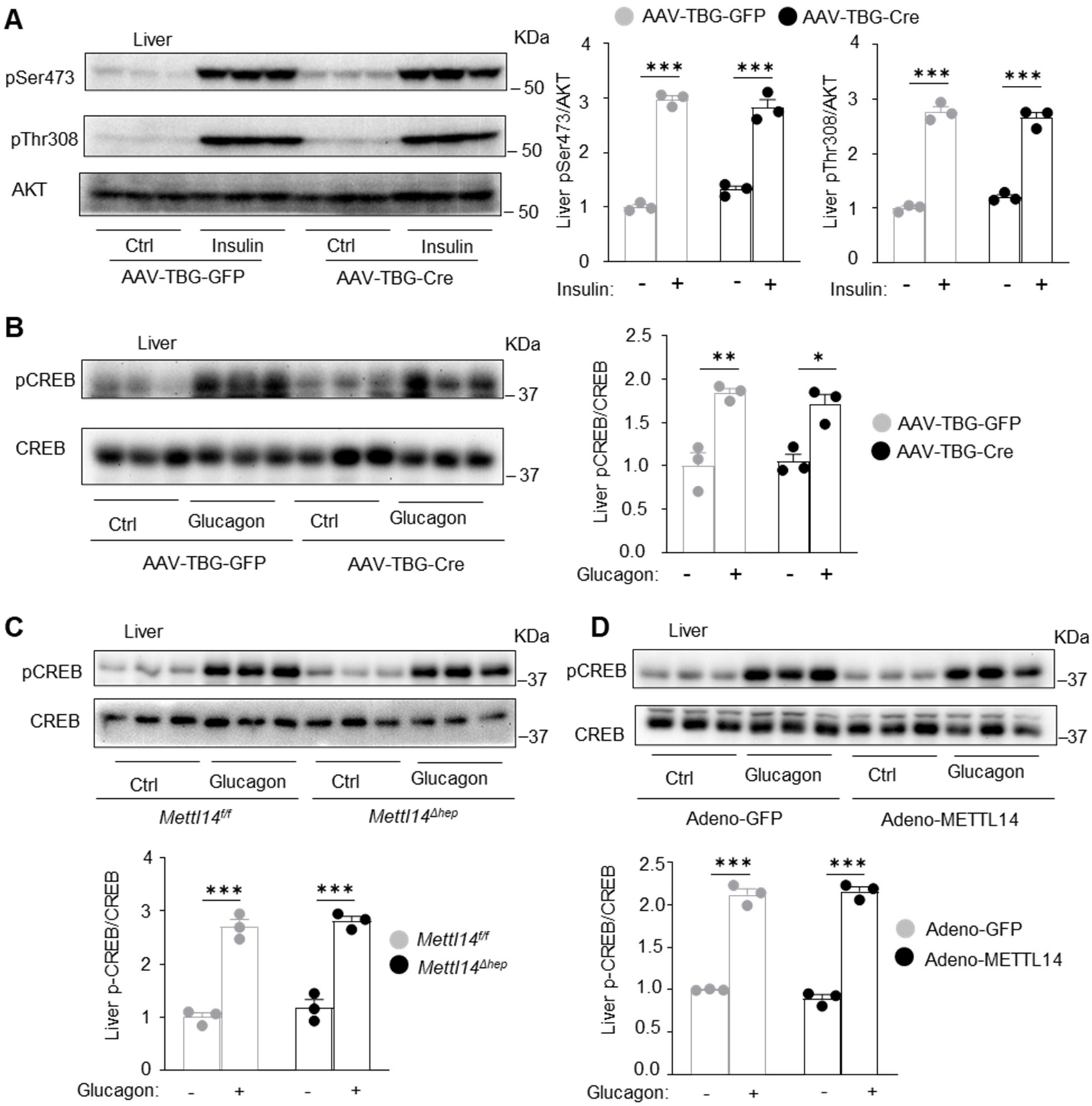

Supplemental Figure 4. METTL14 does not directly alter insulin and glucagon signaling. **(A-B)** *Mettl14$^{f/f}$* male mice were fed HFD for 10 weeks and then transduced with AAV8-TBG-Cre or AAV8-TBG-GFP vectors. Six weeks later (on HFD), mice were fasted overnight and

stimulated with insulin (1 unit/kg) for 5 min or with glucagon (15 μg/kg) for 15 min. Liver extracts were immunoblotted with the indicated antibodies. Phosphorylation of AKT or CREB was normalized to total AKT or CREB levels, respectively (n=3 mice per group). **(C)** *Mettl14*$^{f/f}$ and *Mettl14*$^{\Delta hep}$ mice were fed an HFD for 10 weeks, fasted overnight, and stimulated with glucagon. Liver CREB phosphorylation was assessed by immunoblotting. **(D)** C57BL/6J male mice (on chow diet) were transduced with GFP or METTL14 adenoviral vectors. Two weeks later, mice were fasted overnight and stimulated with glucagon (15 μg/kg) for 15 min. Liver extracts were immunoblotted with the indicated antibodies. Phosphorylation of CREB was normalized to total CREB levels (n=3 mice per group). Data are presented as mean ± SEM. *p<0.05, **p<0.01, ***p<0.001, two-way ANOVA with Šidák's multiple-comparison test.

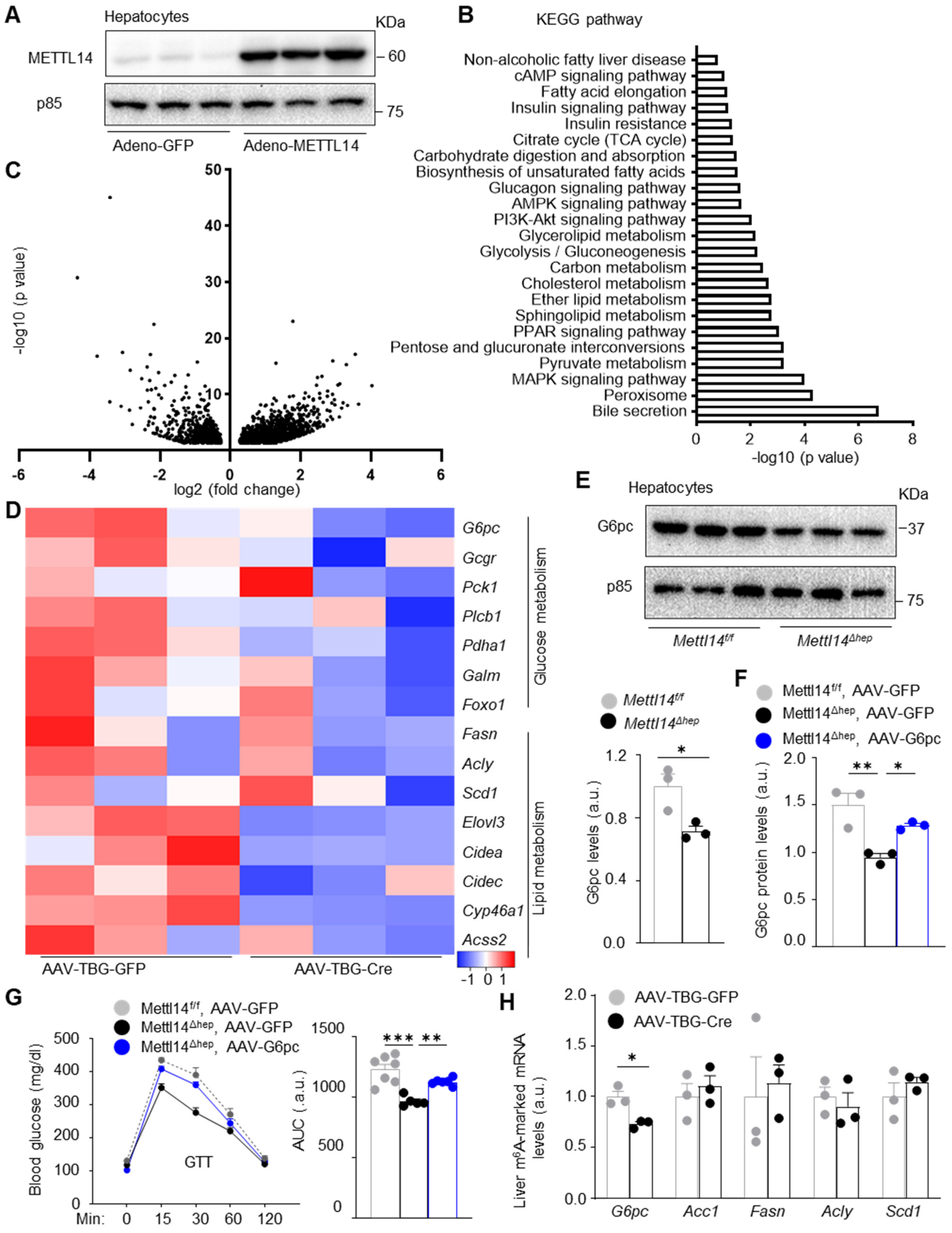

A
Hepatocytes
KDa
METTL14
p85
60
75
Adeno-GFP
Adeno-METTL14
B
KEGG pathway
Non-alcoholic fatty liver disease
cAMP signaling pathway
Fatty acid elongation
Insulin signaling pathway
Insulin resistance
Citrate cycle (TCA cycle)
Carbohydrate digestion and absorption
Biosynthesis of unsaturated fatty acids
Glucagon signaling pathway
AMPK signaling pathway
PI3K-Akt signaling pathway
Glycerolipid metabolism
Glycolysis / Gluconeogenesis
Carbon metabolism
Cholesterol metabolism
Ether lipid metabolism
Sphingolipid metabolism
PPAR signaling pathway
Pentose and glucuronate interconversions
Pyruvate metabolism
MAPK signaling pathway
Peroxisome
Bile secretion
-log10 (p value)
C
-log10 (p value)
log2 (fold change)
D
G6pc
Gcgr
Pck1
Plcb1
Pdha1
Galm
Foxo1
Fasn
Acly
Scd1
Elovl3
Cidea
Cidec
Cyp46a1
Acss2
Glucose metabolism
Lipid metabolism
AAV-TBG-GFP
AAV-TBG-Cre
E
Hepatocytes
KDa
G6pc
p85
37
75
Mettl14f/f
Mettl14Δhep
G6pc levels (a.u.)
F
Mettl14f/f, AAV-GFP
Mettl14Δhep, AAV-GFP
Mettl14Δhep, AAV-G6pc
G6pc protein levels (a.u.)
G
Blood glucose (mg/dl)
GTT
Min:
AUC (a.u.)
H
AAV-TBG-GFP
AAV-TBG-Cre
Liver m6A-marked mRNA levels (a.u.)
G6pc
Acc1
Fasn
Acly
Scd1

Supplemental Figure 5. METTL14 promotes HGP through G6pc. (A) C57BL/6J mouse primary hepatocytes were transduced with METTL14 or GFP adenoviral vectors for 48 h. Cell extracts were immunoblotted with antibodies against METTL14 or p85 (loading control). **(B-D)** *Mettl14*$^{f/f}$ male mice were fed HFD for 10 weeks, and then transduced with AAV8-TBG-Cre (n=3) or AAV8-TBG-GFP vectors (n=3). Eight weeks later, livers were isolated for RNA-seq analysis. **(B)** KEGG pathways based on GO analyses of upregulated and downregulated genes. **(C)** A volcano plot of the upregulated and downregulated genes. *G6pc* transcript was marked. **(D)** Gene expression heatmap. **(E)** Primary hepatocytes were purified from *Mettl14*$^{f/f}$ and *Mettl14*$^{\Delta hep}$ mice at 9 weeks of age. Hepatocyte extracts were immunoblotted with antibodies against G6pc and p85. G6pc levels were normalized to p85 levels (n=3 mice per group). **(F-G).** Males (8 wks old) were transduced with the indicated AAV vectors and fed a normal chow diet. **(F)** Liver extracts were immunoblotted with anti-G6pc antibody. G6pc levels were normalized to p85 levels (n=3 mice per group). **(G)** GTT was performed 4 wks after AAV transduction. *Mettl14*$^{f/f}$, AAV-GFP: n=7, *Mettl14*$^{\Delta hep}$, AAV-GFP: n=5, *Mettl14*$^{\Delta hep}$, AAV-G6pc: n=6. AUC: area under curve. a.u.: arbitrary unit. **(H)** *Mettl14*$^{f/f}$ males were fed a HFD for 10 weeks and then transduced with AAV8-TBG-GFP or AAV8-TBG-Cre vectors. Six weeks later, m^6A levels in *G6pc*, *Acc1*, *Fasn*, *Acly* and *Scd1* transcripts were measured in the liver using m^6A-RIP (n=3 mice per group). Data are presented as mean ± SEM. *p<0.05, **p<0.01, ***p<0.001, two-sided unpaired *t*-test (**E**, **H**) and one-way ANOVA with Tukey's multiple-comparison test (**F**-**G**).

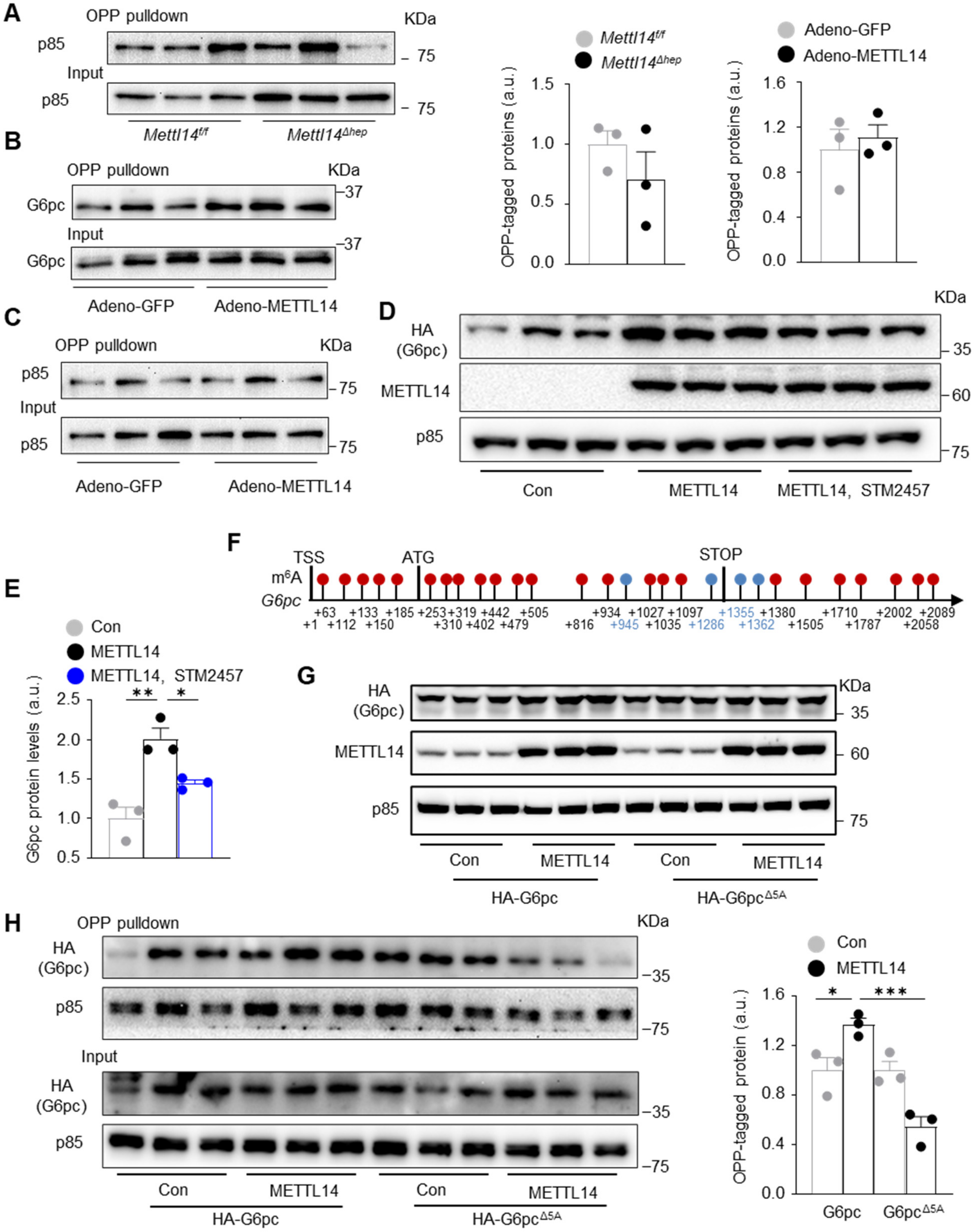

Supplemental Figure 6. METTL14 m^{6}A-dependently increases G6pc biosynthesis. (A) Primary hepatocytes were isolated from *Mettl14$^{f/f}$* and *Mettl14$^{\Delta hep}$* males at 8 weeks of age. Newly-synthesized and OPP-tagged p85 protein was measured by anti-p85 antibody in OPP

assays and normalized to p85 input (n=3 mice per group). **(B)**. Primary hepatocyte culture (C57BL/6J males) was transduced with METTL14 or GFP adenoviral vectors for 24 h and subjected to OPP assays (normalized to G6pc input, n=3 per group). **(C)** Primary hepatocyte cultures were prepared from C57BL/6J males and transduced with METTL14 or GFP adenoviral vectors for 24 h. Newly-synthesized and OPP-tagged p85 protein was measured by anti-p85 antibody in OPP assays and normalized to p85 input (n=3 mice per group). **(D-E)** Huh7 hepatocytes were cotransfected with *METTL14* and *G6pc* plasmids. 12 h later, cells were treated with STM2457 (5 µg/ml) (DMSO as control) for 36 h. Cell extracts were immunoblotted with anti-HA antibody. HA-G6pc levels were normalized to p85 levels (n=3 per group). **(F)** The m^6A sites in *G6pc* mRNA. The number indicate the m^6A position (TSS: +1). Blue color shows the mutated m^6A in $G6pc^{\Delta 5A}$ mRNA. TSS: transcription start site. **(G)** Huh7 hepatocytes were cotransfected with *METTL14* and *G6pc or* $G6pc^{\Delta 5A}$ plasmids for 2 days, and cell extracts were immunoblotted with the indicated antibodies. **(H)** Huh7 hepatocytes were cotransfected with *METTL14* and *HA-G6pc or* $HA\text{-}G6pc^{\Delta 5A}$ plasmids. 36 h later, OPP assays were performed to measure G6pc translation. OPP-marked G6pc levels were normalized to inputs (n=3 per group). Data are presented as mean ± SEM. *p<0.05, **p<0.01, ***p<0.001, one-way ANOVA with Tukey's multiple-comparison test.

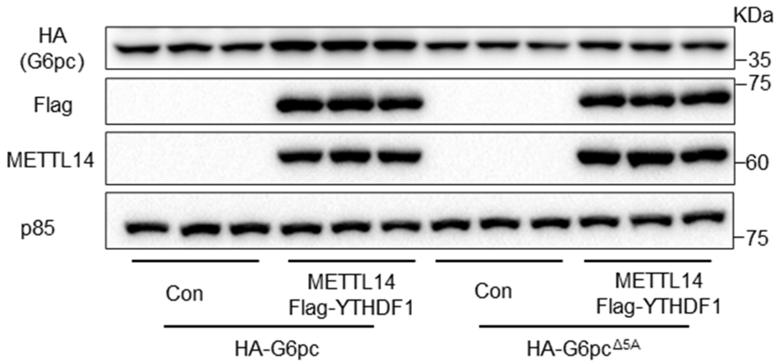

Supplemental Figure 7. YTHDF1 m^6A-dependently increases G6pc synthesis. Huh7 cells were cotransfected with *METTL14*, *YTHDF1* and *HA-G6pc* or $HA\text{-}G6pc^{\Delta 5A}$ plasmids for 2 days, and cell extracts were immunoblotted with the indicated antibodies.

Target gene	Modification type	Genomic location	Source	Support datasets
G6pc	m6A	chr11:101258448(+)	RMBase	GSM908344
G6pc	m6A	chr11:101258497(+)	RMBase	GSM908344
G6pc	m6A	chr11:101258518(+)	RMBase	GSM908344
G6pc	m6A	chr11:101258535(+)	RMBase	GSM908344
G6pc	m6A	chr11:101258570(+)	RMBase	GSM1828595, GSM908344

G6pc	m6A	chr11:101258638(+)	RMBase	GSM1828595, GSM908344
G6pc	m6A	chr11:101258695(+)	RMBase	GSM1828595, GSM908344
G6pc	m6A	chr11:101258704(+)	RMBase	GSM1828595, GSM908344
G6pc	m6A	chr11:101258787(+)	RMBase	GSM908344
G6pc	m6A	chr11:101258827(+)	RMBase	GSM908344
G6pc	m6A	chr11:101261543(+)	RMBase	GSM908344
G6pc	m6A	chr11:101261569(+)	RMBase	GSM908344
G6pc	m6A	chr11:101267130(+)	RMBase	GSM908344
G6pc	m6A	chr11:101267248(+)	RMBase	GSM1828595, GSM908344
G6pc	m6A	chr11:101267259(+)*	RMBase	GSM1828595, GSM908344
G6pc	m6A	chr11:101267341(+)	RMBase	GSM1828595, GSM908344
G6pc	m6A	chr11:101267349(+)	RMBase	GSM1828595, GSM908344
G6pc	m6A	chr11:101267411(+)	RMBase	GSM908344
G6pc	m6A	chr11:101267600(+)*	RMBase	GSM1828595, GSM908344
G6pc	m6A	chr11:101267670(+)*	RMBase	GSM1828595, GSM908344
G6pc	m6A	chr11:101267677(+)*	RMBase	GSM1828595, GSM908344
G6pc	m6A	chr11:101267695(+)	RMBase	GSM1828595, GSM908344
G6pc	m6A	chr11:101267820(+)	RMBase	GSM1828595, GSM908344
G6pc	m6A	chr11:101268025(+)	RMBase	GSM1828595
G6pc	m6A	chr11:101268102(+)	RMBase	GSM1828595
G6pc	m6A	chr11:101268317(+)	RMBase	GSM1828595, GSM908344
G6pc	m6A	chr11:101268373(+)	RMBase	GSM1828595, GSM908344
G6pc	m6A	chr11:101268404(+)	RMBase	GSM1828595, GSM908344

Table S1. Liver m^{6}A-seq datasets and RM2Target analysis. * Also identified by the SRAMP Prediction Server.

**ANTIBODY**	**SOURCE**	**Cat#**	**Blot**
METTL3	ABclonal	A8370	1:2000
METTL14	Sigma	HPA038002	1:2000
WTAP	ABclonal	A14695	1:1000

m^6A	Cell Signaling Technology	56593	1:2000
pAKT (pThr308)	Cell Signaling Technology	2965	1:2000
pAKT (pSer473)	Cell Signaling Technology	4060	1:2000
AKT	Cell Signaling Technology	2920	1:2000
pCREB	Cell Signaling Technology	9198	1:2000
CREB	Cell Signaling Technology	4820	1:2000
G6PC	ABclonal	A21168	1:1000
p85	Home made	N/A	1:5000
Lamin A/C	Cell Signaling Technology	4777	1:2000
ACC1	Cell Signaling Technology	3676	1:2000
FASN	Cell Signaling Technology	3180	1:2000
ACLY	Cell Signaling Technology	4332	1:2000
SCD1	Cell Signaling Technology	2794	1:2000
HA	Home made	N/A	1:2000
Flag	Sigma	F1804	1:5000
FTO	Abcam	Ab94482	1:2000
ALKBH5	Proteintech Group	16837-1-AP	1:1000
YTHDF1	ABclonal	A23773	1:2000
YTHDF2	Cell Signaling Technology	71283	1:2000
YTHDF3	ABclonal	A8395	1:2000

Table S2. Antibody list

**Genes**	**Forward**	**Reverse**
Mettl3	AGCAGGACTCTGGGCACTT	GCTTAGGGCCGCTAGAGGTA
36B4	AAGCGCGTCCTGGCATTGTCT	CCGCAGGGGCAGCAGTGGT
Mettl14	GCTTGCGAAAGTGGGGTTAC	AATGAAGTCCCCGTCTGTGC
Wtap	GCTTTGGAGGGAAAGTACAC	CATCTCCTGCTCTTTGGTTG
Fto	AGAACCTGGTGGACAGGTCA	CTGGTGTCTCGATGTCCCAA
Alkbh5	CTTTGCTTCGGCTGCAAGTT	AATGTCCTGAGGCCGTATGC
Acc1	CAGGGACTATGTCCTGAAGCA	GGAATCCATTGTGGAGAGGA
Fasn	TTGACGGCTCACACACCTAC	CGATCTTCCAGGCTCTTCAG
Acly	CCTCAAGGACTTCGTCAAACA	GCCCATACTCCTTCCTAGCAC
Scd1	AGGTGCCTCTTAGCCACTGA	CCAGGAGTTTCTTGGGTTGA
G6pc	CCGGTGTTTGAACGTCATCT	CAATGCCTGACAAGACTCCA
Gcgr	CACCCTCTGCCCAGGTAATG	GCAGGAAATGTTGGCAGTGG
Pck1	ATCATCTTTGGTGGCCGTAG	ATCTTGCCCTTGTGTTCTGC
Pdk4	GCTTGCCAATTTCTCGTCTC	CCTGCTTGGGATACACCAGT

**Cloning Primers**	**Sequences**
G6pc$^{\Delta 5A}$-1F	TCTACAATGCCAGCCTCCGGAAGTATTGTCTCATCACCATCTTCTT
G6pc$^{\Delta 5A}$-2R	GTGTGACTGACCCAGGATCCGGGCTAGGC
G6pc$^{\Delta 5A}$-3F	GGATCCTGGGTCAGTCACACAAGAAGTCTTTGTA
G6pc$^{\Delta 5A}$-4R	TTGATCCTAGACCTTTGCATGGCGGTTGAC
G6pc$^{\Delta 5A}$-5F	ATGCAAAGGTCTAGGATCAACTAAAGCCTCTGAAAC

G6pc$^{\Delta 5A}$-6R	ACAGTGTGATTTTTATGTACAGTGGAGACTATCTGGAAGCAG
G6pc$^{\Delta 5A}$-7F	CTCCTGTGGTCTTTGGAGAAAGCTAAGAGATGGTG
G6pc$^{\Delta 5A}$-8R	TTCTCCAAAGACCACAGGAGGTCCACCCCTAG
G6pc-cloning-F	CAGCGGATCCACTAGTATGGAGGAAGGAATGAACATTCTCC
G6pc-cloning-R	GATTGGATCCAAGCTTGTGCTTGGTGTGGGTGAA
YTHDF1-cloning-F	CAGCGGATCCACTAGTATGTCGGCCACCAGCGTG
YTHDF1-cloning-R	TCGATAAGCTCTCGAGTCATTGTTTGTTTCGACTCTGCCG
YTHDF3-cloning-F	CAGCGGATCCACTAGTATGTCAGCCACTAGCGTGG
YTHDF3-cloning-R	TCGATAAGCTCTCGAGTTATTGTTTGTTTCTATTTCTCTCCCTAC

Table S3. Primer list
